# Zedoary turmeric oil injection ameliorates lung inflammation via platelet factor 4 and regulates gut microbiota disorder in respiratory syncytial virus-infected young mice

**DOI:** 10.1186/s13020-024-00954-6

**Published:** 2024-06-11

**Authors:** Yu-Zhuo Wu, Qian Zhang, Hua Li, Cheng-Xi Jiang, Xiao-Kun Li, Hong-Cai Shang, Sheng Lin

**Affiliations:** 1 Key Laboratory of Chinese Internal Medicine of Ministry of Education and Beijing, Dongzhimen Hospital, Beijing University of Chinese Medicine, Beijing, 100700 China; 2 School of Pharmacy, Wenzhou Medical University, Wenzhou, 325035 China; 3 Wuya College of Innovation, Key Laboratory of Structure-Based Drug Design and Discovery, Ministry of Education, Shenyang Pharmaceutical University, Shenyang, 110016 China

**Keywords:** Traditional Chinese medicine, Zedoary turmeric oil injection, Respiratory syncytial virus pneumonia, Platelet factor 4

## Abstract

**Background:**

Respiratory syncytial virus (RSV)-induced lung inflammation is one of the main causes of hospitalization and easily causes disruption of intestinal homeostasis in infants, thereby resulting in a negative impact on their development. However, the current clinical drugs are not satisfactory. Zedoary turmeric oil injection (ZTOI), a patented traditional Chinese medicine (TCM), has been used for clinical management of inflammatory diseases. However, its in vivo efficacy against RSV-induced lung inflammation and the underlying mechanism remain unclear.

**Purpose:**

The present study was designed to confirm the in vivo efficacy of ZTOI against lung inflammation and intestinal disorders in RSV-infected young mice and to explore the potential mechanism.

**Study design and methods:**

Lung inflammation was induced by RSV, and cytokine antibody arrays were used to clarify the effectiveness of ZTOI in RSV pneumonia. Subsequently, key therapeutic targets of ZTOI against RSV pneumonia were identified through multi-factor detection and further confirmed. The potential therapeutic material basis of ZTOI in target tissues was determined by non-target mass spectrometry. After confirming that the pharmacological substances of ZTOI can reach the intestine, we used 16S rRNA-sequencing technology to study the effect of ZTOI on the intestinal bacteria.

**Results:**

In the RSV-induced mouse lung inflammation model, ZTOI significantly reduced the levels of serum myeloperoxidase, serum amyloid A, C-reactive protein, and thymic stromal lymphoprotein; inhibited the mRNA expression of IL-10 and IL-6; and decreased pathological changes in the lungs. Immunofluorescence and qPCR experiments showed that ZTOI reduced RSV load in the lungs. According to cytokine antibody arrays, platelet factor 4 (PF4), a weak chemotactic factor mainly synthesized by megakaryocytes, showed a concentration-dependent change in lung tissues affected by ZTOI, which could be the key target for ZTOI to exert anti-inflammatory effects. Additionally, sesquiterpenes were enriched in the lungs and intestines, thereby exerting anti-inflammatory and regulatory effects on gut microbiota.

**Conclusion:**

ZTOI can protect from lung inflammation via PF4 and regulate gut microbiota disorder in RSV-infected young mice by sesquiterpenes, which provides reference for its clinical application in RSV-induced lung diseases.

**Supplementary Information:**

The online version contains supplementary material available at 10.1186/s13020-024-00954-6.

## Introduction

Respiratory syncytial virus (RSV), one of the leading causes of childhood mortality in infants below 6 months of age, results in substantial public health burden in low- and middle-income countries (LMICs) [1]. In 2019, 33 million cases of acute lower-respiratory tract infections caused by RSV infection occurred worldwide in children under the age of 5 years, with approximately 10% requiring hospitalization and resulting in approximately 100,000 deaths (case fatality rate of 0.3%). The gut microbiota of children are extremely fragile, and RSV infection can disrupt the gut microbiota, causing adverse effects on the children’s growth and development. However, this issue is often overlooked in clinical practice [2]. Considering the limited therapeutic resources for RSV infections, it is critical to develop new interventions and inform decision-makers about their effectiveness [3].

Ethnic medicines derived from plants have played an indelible role in the history of disease treatment worldwide. In long-term clinical practice, phytotherapeutic strategies have formed a unique theoretical system and accumulated valuable clinical experience [4]. Zedoary turmeric oil injection (ZTOI), a patented traditional Chinese medicine (TCM) and the volatile oil component extracted from Curcuma zedoaria, has been used against pneumonia caused by multiple factors in clinical practice in China [5]. Our previous research has shown that ZTOI contains more than 11 main components, including curcumene, germacrone, curdione, neocurdione, and curcumenol [6], most of which can attenuate inflammation. For example, curcumol, the most extensively researched ingredient in zedoary turmeric oil (ZTO), can inhibit the JAK1/STAT3 signaling pathway to exert anti-inflammatory activity [7]. ZTOI can also alleviate lipopolysaccharides-induced acute lung injury in rats by multiple anti-inflammatory mechanisms [6]. Another study has shown that ZTO can protect the L02 cells against acute liver injury by regulating the PI3K/Akt/FoxO1 pathway, as well as restore the damaged mitochondria function and redox imbalance [8]. Therefore, ZTOI and its components have important value in the treatment of inflammatory diseases. In addition, ZTO exhibits extensive antiviral activities against influenza virus, adenovirus, H1N1, and rotavirus [9, 10]. Germacrone has shown in vitro antiviral effects on influenza [11]. Therefore, ZTO has important potential value in the treatment of viral pneumonia, but currently there is a lack of in vivo pharmacodynamic research.

Platelet factor 4 (PF4), also known as CXCL4, has long been considered a founding member of the C-X-C chemokine family that is only expressed in mature platelets. Previous studies have shown that PF4 can also be produced in macrophages and activated T cells, but its specific function has not been elucidated [12, 13]. Early studies on PF4 have mainly focused on its anticoagulant function [14]. Recently, relevant studies have shown that PF4 has a wide range of biological functions in the occurrence, development, and treatment of inflammatory diseases, including promoting neutrophil adhesion, regulating megakaryocyte maturation, inducing monocyte differentiation into macrophages [15], maintaining helper T-cell differentiation [16], maintaining hematopoietic stem cell quiescence [17], and promoting B-cell differentiation in the bone marrow environment [18]. However, it is worth noting that PF4 may have completely opposite regulatory patterns in different target organs and physiological environments, thereby promoting or inhibiting the development of inflammation [19, 20]. Therefore, the function of PF4 in inflammatory diseases deserves in-depth research, and PF4 may become a new target for disease treatment.

The aim of our study was to confirm the in vivo efficacy of ZTOI against lung inflammation and intestinal disorders in RSV-infected young mice, and to explore the potential mechanism. Based on an RSV-induced mouse lung inflammation model and cytokine antibody arrays technology, we first confirmed the in vivo efficacy of ZTOI against RSV-induced lung inflammation. Subsequently, the key therapeutic targets of ZTOI against RSV-induced lung inflammation were identified through multifactor detection and were further confirmed by western blotting, real-time reverse transcription–polymerase chain reaction, and immunofluorescence. Subsequently, the potential therapeutic material basis of ZTOI in target tissues was determined by non-target mass spectrometry. After confirming that the pharmacological substances of ZTOI can reach the intestine, we used 16S rRNA-sequencing technology to study the effect of ZTOI on the intestinal bacteria. Our results showed that ZTOI can protect from lung inflammation via PF4 and regulate gut microbiota disorder in RSV-infected young mice by sesquiterpenes, which provides reference for the clinical application of ZTOI for RSV-induced lung inflammation.

## Materials and methods

### Reagents and materials

ZTOI (batch numbers: 4190401, 4190603, and 4200603; 5 mL: 50 mg per bottle) was provided by Hefei Future Drug Development Co., Ltd. (Hefei, China). Ribavirin injection (batch number: 2101290642; 2 mL: 0.1 g) was purchased from Cisen Pharmaceutical Co., Ltd. (Shandong, China). BALB/c mice weighing between 8 and 10 g were purchased from Beijing Vital River Laboratory Animal Technology (Beijing, China). Myeloperoxidase (MPO, cat #DG30347M-96T), serum amyloid A (SAA, cat #DG30419M-96T), C-reactive protein (CRP, #DG30086M-96T), and thymic stromal lymphoprotein (TSLP, cat #DG91371Q-96T) ELISA kits were purchased from Beijing Dogesce Biotechnology Co., Ltd. (Beijing, China). Anti-RSV antibody (anti-RSV, cat #ab43812) was obtained from Beijing Dogesce Biotechnology Co., Ltd. (Beijing, China). Anti-PF4 antibody (cat #A17376) was purchased from Wuhan ABclonal Technology Co., Ltd. (Wuhan, China). SuperReal PreMix Plus (SYBR Green, cat #FP205-02), FastKing cDNA First Strand Synthesis Kit (cat #KR116-02), and Total RNA Extraction Kit (Centrifuge Column Type, cat #DP419) were purchased from Tiangen Biotech (Beijing) Co., Ltd. (Beijing, China). RSV virus strain was purchased from ATCC (#VR-1540), propagated and quantified on Hep-2 cell, then stored at − 80 ℃. Virus titers were determined using a methylcellulose plaque assay.

### Animal experiments

Specific pathogen free (SPF) BALB/c male mice weighing 9–10 g were fed for one week before the experiments. The mice were randomly divided into five groups, with 10 animals in each group, including the control group, the RSV group, the ribavirin group, the ZTOI-L group (clinical equivalent dose of ZTOI), and the ZTOI-H group (twice the clinical equivalent dose). In every group excepting the control group, the mice were intranasally inoculated with 50 μL of RSV (1 × 10^6^ PFU/mL, 100TCID_50_) after anesthesia with isoflurane. Viral stock was diluted with phosphate buffer saline (PBS). The control group was treated with an equal volume of physiological saline. After 30 min, the ribavirin group was treated with ribavirin 100.0 mg/kg/day. The ZTOI-L and ZTOI-H groups were administered with ZTOI at 61.5 mg/kg/day and 123.0 mg/kg/day, respectively. The control and RSV groups were all given an equal volume of physiological saline. The aforementioned drug delivery routes were all via tail vein injection. Drug interventions lasted for 5 days. On the third day, RSV virus solution was replenished in all groups except the control group to ensure the successful induction of an RSV-induced pneumonia model. Anesthesia for material collection was performed on the sixth day by sodium pentobarbital (*w*/*w* 1%). Blood samples were collected from an eyeball. Parts of lung tissues were stored and fixed with 4% neutral-buffered formalin for subsequent histological examination, and other parts of lung tissues and feces were stored at − 80°C for subsequent experiments. All experiments were supported by the Institute of Chinese Materia Medica, China Academy of Chinese Medical Sciences (registration and ethical approval number: 2021D124). Animal use and care followed the ARRIVE guidelines 2.0 [21].

### Calculation of the lung index

After obtaining the body weight of each mouse (n = 10), the lung tissues were immediately excised, cleaned in physiological saline, dehydrated with a filter paper, and weighed. The lung index was calculated using formula I to assess the magnitude of pulmonary edema.

1 $$\text{Lung index}\text{ = }\frac{{\text{Lung}} \, {\text{weight}}(mg)}{{\text{Body}} \, {\text{weight}}(g)} \times 100\%$$

### Histopathological evaluation of lung tissues

The lung tissues of all groups were embedded in paraffin and then cut into 4-μm-thick sections after 24 h fixation in 4% neutral-buffered formalin. The sections were stained with hematoxylin and eosin (HE) and then evaluated under a screen digital microscope. Semiquantitative evaluation was performed as described in Table 1. At least three pathological films were taken from each group, and each pathological film needed to have at least three visual fields taken for scoring. Pulmonary parenchymal area was measured by Image Pro Plus 6.0. Lung parenchymal area (%) was calculated as follows: area of lung parenchyma/total lung area × 100%.

**Table 1:** Semiquantitative evaluation of lung tissue sections

Score	Criteria
0	The alveolar wall is intact without thickening, inflammatory infiltration, or congestion
1	Mild diffuse inflammatory cell infiltration (neutrophils) in the alveolar wall, without thickening of the alveolar wall
2	Significant extensive infiltration of inflammatory cells (neutrophils and monocytes), with slight thickening of the alveolar walls (1–2 times)
3	Severe inflammatory cell infiltration with 2–threefold thickening of the alveolar walls in individual areas
4	Severe inflammatory cell infiltration and significant thickening of the alveolar walls in 25–50% of the parenchyma of lung tissue
5	Severe inflammatory cell infiltration and significant thickening of the alveolar walls in more than 50% of the parenchyma of lung tissue

### MPO, SAA, CRP, and TSLP measurements

The levels of MPO, SAA, CRP, and TSLP in both serum and lung tissue samples were measured in accordance with the manufacturer's instructions using ELISA kits.

### Immunofluorescence

Immunofluorescence (IF) staining was performed by the tyramide signal amplification (TSA) method. Briefly, the lung samples were treated with primary antibodies, including PF4 and RSV, and then incubated with specific secondary antibodies. After washing, the slides were incubated with the fluorescence reagent and then heated to remove the combined antibodies. The final image was acquired with the excitation wavelengths of DAPI channel (blue), Cy3 channel (red), and Cy5 channel (pink).

### Quantitative measurement of 40 mouse cytokines

The protein microarray slide was purchased from RayBiotech with the product number QAM-INF-1. The samples were diluted in accordance with the instructions. A total of 100 µL of sample diluent was added to each sample well, and subsequently incubated on a shaker for 1 h (room temperature) in order to seal the antibody. Then, the buffer solution was extracted from each well, and 60 µL of standard solution and diluted sample solution was added to the well and incubated in a horizontal shaker at 4°C overnight. After using a chip washing machine to clean the glass slide in accordance with the instructions, we centrifuged the antibody mixture, and then used a pipette to add 1.4 mL of sample diluent to the small tube. After blowing evenly, centrifuging quickly, and adding 80 µL detection antibody per well, the tube was incubated on a horizontal shaker for 2 h (temperature 37°C). We centrifuged the Cy3-Streptomyces avidin small tube, added 1.4 mL of sample diluent to the tube with a pipette, blew evenly, and centrifuged it quickly. We added 80 µL of Cy3-Streptomyces avidin per well, and incubated the slide in dark at 37°C for 1 h. Finally, a laser scanner, InnoScan 300 Microarray Scanner, was applied for signal scanning. The data screening and analysis were completed based on QAM-INF-1 corresponding data analysis software.

### Quantitative real-time PCR (qPCR)

Total RNA from the lung tissue samples was extracted using TRIzol reagent and NucleoSpin RNA clean-up kit (Macherey–Nagel, Duren, Germany). Complementary DNA (cDNA) was generated using a FastKing cDNA First Strand Synthesis Kit. The relative expression levels of the specific genes were determined with an Alilent Real-Time PCR system. The data were calculated by the cycle threshold (ΔΔCT) method and normalized to β-actin. The primers of the target gene were designed as shown in Table 2.

**Table 2:** Primer sequence

Gene	Forward primer	Reverse primer
β-Actin	AAGGCCAACCGTGAAAAGAT	GTGGTACGACCAGAGGCATAC
PF4	TGTGAAGACCATCTCCTC	GCTGATACCTAACTCTCCA
IL-6	AGCCAGAGTCCTTCAGAGAGA	GGATGGTCTTGGTCCTTAGCC
IL-1β	CAGCTGGAGAGTGTGGATCC	TGCTTGTGAGGTGCTGATGT
TNF-α	TCTTCAAGGGACAAGGCTGC	CTCCAAAGTAGACCTGCCCG
RSV	TGATACACTSAACAAAGATCAACTTCTG	TCTCCTGTGCTMCGTTGRAT

### Western blotting

To perform western blotting, the lung tissue samples were washed twice with ice-cold phosphate-buffered saline (PBS) and lysed in RIPA buffer supplemented with protease inhibitor cocktail (Roche, CH). The proteins were separated by SDS-PAGE and transferred to a PVDF (Sigma-Aldrich) membrane. The membrane was blocked with 5% skim milk in tris-buffered saline with 0.1% tween 20 (TBS-T) buffer for 2 h and then immunoblotted with primary antibodies at 4°C overnight. Next, horse radish peroxidase (HRP)-conjugated secondary antibodies were added to the membrane and incubated at 25℃ for 2 h. Finally, the membrane was visualized by a Tanon 4600 system (Tanon, Shanghai, China).

### Screening of the potential therapeutic material basis of ZTOI in target tissues

BALB/c mice weighing 9–10 g were treated with ZTOI at a single dosage of 123.0 mg/kg. The operation method of blood, lung, and feces collections was the same as described in Animal experiments. The preparation of analytical samples and data collection were done in line with previous work [6].

### Analysis of fecal 16S rRNA

Sterile EP tubes were used to collect 5–7 feces from each group, and the collected feces were immediately stored in liquid nitrogen. The DNA samples of fecal microbial community genomic were extracted using the E.Z.N.A.® soil DNA Kit and checked by NanoDrop 2000 UV–vis spectrophotometer (Thermo Scientific, Wilmington, USA). The hypervariable region V3–V4 of the bacterial 16S rRNA gene was amplified with primer pairs 338F (5ʹ-ACTCCTACGGGAGGCAGCAG-3ʹ) and 806R (5ʹ-GGACTACHVGGGTWTCTAAT-3ʹ) by an ABI GeneAmp® 9700 PCR thermocycler (ABI, CA, USA). The PCR product was extracted from 2% agarose gel and purified using the AxyPrep DNA Gel Extraction Kit in accordance with the manufacturer’s instructions and quantified using Quantus™ Fluorometer (Promega, USA).

The purified amplicons were pooled in equimolar and paired-end sequenced on an Illumina MiSeq PE300 platform in line with the standard protocols by Majorbio Bio-Pharm Technology Co. Ltd. (Shanghai, China).

The raw 16S rRNA gene-sequencing reads were demultiplexed and quality-filtered by fast version 0.20.0 [22] and merged by FLASH version 1.2.7 [23]. Operational taxonomic units (OTUs) with a 97% similarity cutoff were clustered using UPARSE version 7.1 [24], and chimeric sequences were identified and removed. The taxonomy of each OTU representative sequence was analyzed by RDP Classifier version 2.2 against the 16S rRNA database (eg. Silva v138) using a confidence threshold of 0.7 [25].

### Statistical approaches

All the experiments were repeated at least three times. The data were presented as the mean ± standard error of the mean (SEM) and analyzed using one-way ANOVA to determine significant differences between multiple groups. Results were considered statistically significant at *P* < 0.05.

## Results

### ZTOI improves serum inflammatory markers in young mice with RSV-induced lung inflammation

Considering the intravenous administration of ZTOI and the drug tolerance of young mice in our experiments, non–RSV-infected young mice were first injected with a double dose of ZTOI through the tail vein to rule out the death and inflammation caused by excessive drug use. As shown in Supporting information Fig. S1, after administering twice the clinical equivalent dose of ZTOI (123.0 mg/kg/day) for 5 days, there were no abnormalities in the morphology and structure of the heart, liver, spleen, lungs, and kidneys of the treated mice compared with the untreated group (Supporting information Fig. S1). Additionally, the inflammatory markers such as MPO, SAA, CRP, and TSLP in the lungs and serum were measured to evaluate the effects of ZTOI in healthy mice. Consistently, there were no significant changes of the four indicators in the lungs and serum (Supporting information Fig. S2). The increase of SAA and CRP in blood might be caused by prolonged and excessive injection of drugs into the tail vein (Supporting information Fig. S2 b2 and b3). Therefore, we designated the high-dose group as twice the clinical equivalent dose in our study.

**Fig. 1:**
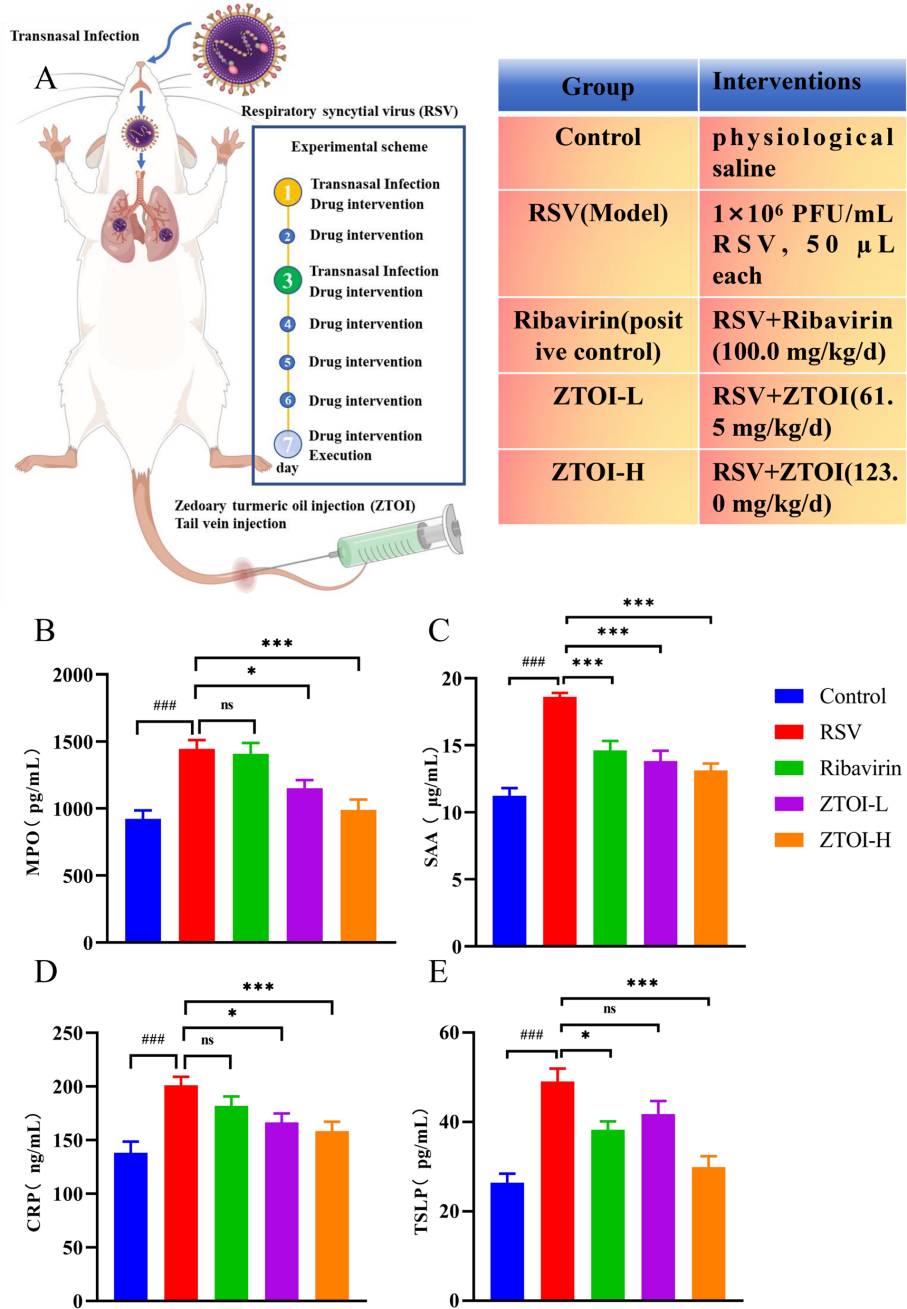
Pharmacodynamic evaluation diagram (**A**) and changes of key inflammatory indicators in serum (**B**: myeloperoxidase, MPO; **C**: serum amyloid A, SAA; **D**: C-reactive protein, CRP; **E**: thymic stromal lymphoprotein, TSLP; n = 10; * P < 0.05, *** P < 0.001, compared with the RSV group; ### P < 0.001, compared with the control group; data presented as mean ± standard error of mean)

Nasal instillation is a classic method for establishing an RSV infection model in the lungs [26], as shown in Fig. 1A. MPO, an enzyme that exists in leukocytes, can immediately respond to RSV infection [27]. After infection, MPO in mouse serum increased significantly, while ZTOI inhibited its generation in a dose-dependent manner, as shown in Fig. 1B. SAA, a sensitive marker of respiratory infection, is useful to explore host response to the respiratory infections in clinical research, especially for RSV [28]. ZTOI exhibited better inhibitory activity against SAA production than positive drug ribavirin (Fig. 1C). In addition, ZTOI exhibited dose-dependent inhibitory effects in regulating CRP and TSLP generation in serum (Fig. 1D and E). CRP, one of the systemic inflammatory markers for RSV infection in children, performs better than other blood parameters as a marker [29]. Persistent production of TSLP after early-life RSV infection plays an important role in immune-phenotype alteration, inflammatory-cell accumulation, and allergic-disease enhancement [30, 31]. Therefore, the improvement effects on the abovementioned four critical serum inflammatory markers indicate that ZTOI possesses potential therapeutic and prognostic recovery effects on RSV-induced pneumonia.

### ZTOI alleviates lung tissue inflammatory factors and pathological injury in young mice with RSV-induced pneumonia

**Fig. 2:**
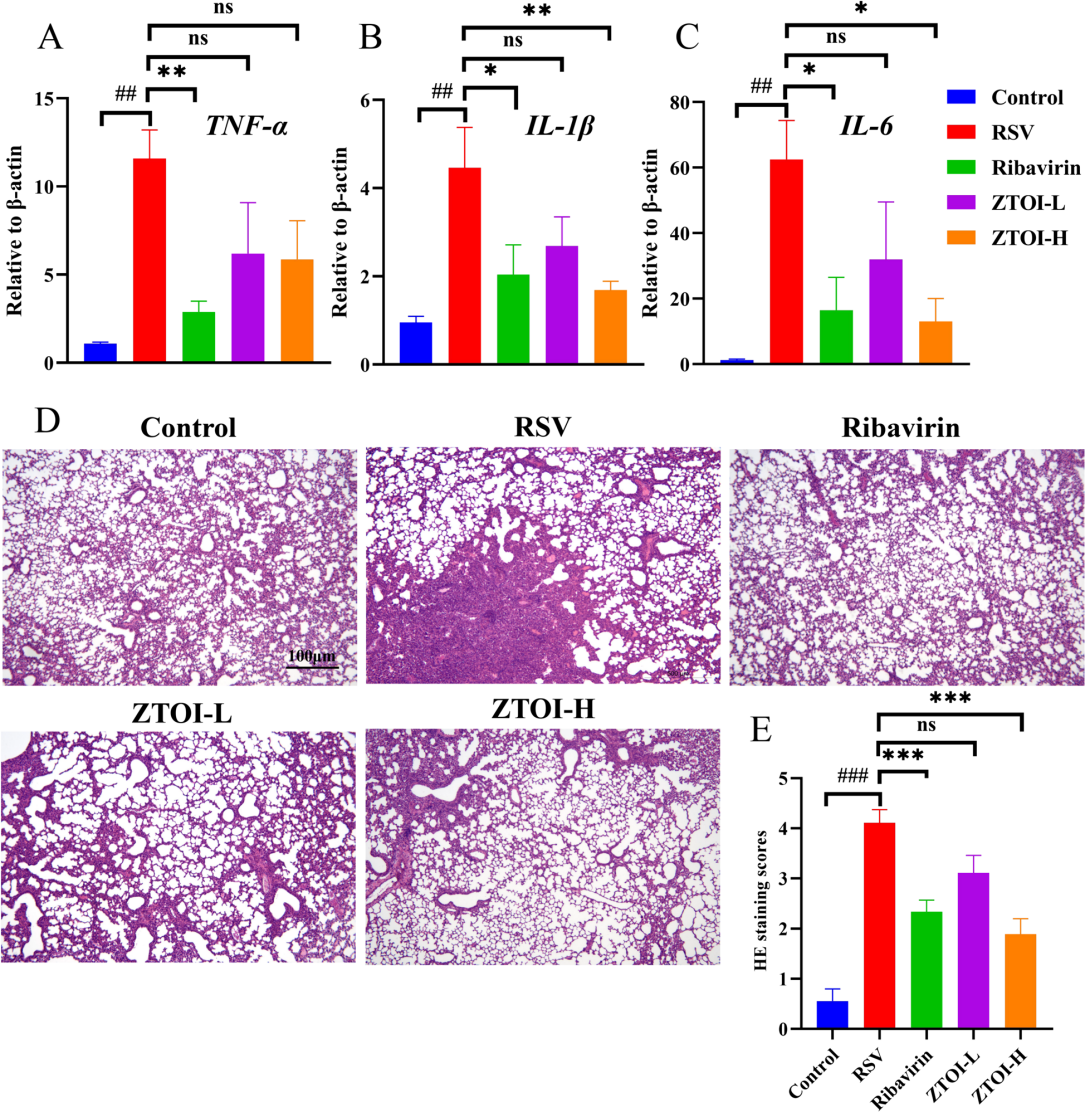
Changes of key inflammatory indicators in lung tissues (**A**: RNA expression level of tumor necrosis factor-α; **B**: RNA expression level of interleukin-1β; **C**: RNA expression level of interleukin-6; n > 3; * P < 0.05, ** P < 0.01, compared with the RSV group; ## *P* < 0.01, compared with the control group; data presented as mean ± standard error of mean) and pathology evaluation (**D**: representative histological images of hematoxylin and eosin–stained lung section; **E**: hematoxylin–eosin score, n = 9; *** P < 0.001, compared with the RSV group; ### P < 0.001, compared with the control group; data presented as mean ± standard error of mean)

To evaluate the inflammatory changes in the lung tissues, we first detected the RNA expression levels of TNF-α, IL-1β, and IL-6 in the lung tissues by real-time fluorescence quantitative PCR. ZTOI inhibited the transcription of TNF-α in the RSV-infected lung tissues, but without reaching statistical significance (Fig. 2A). However, ZTOI exerted dose-dependent and significant inhibition of IL-1β and IL-6 RNA expression in the lung tissues, indicating that ZTOI could prevent lung inflammation caused by RSV (Fig. 2B and C). Consistent with the PCR results, the lungs from the RSV-treated mice displayed severe histopathological changes (Fig. 2D, top-middle), including pulmonary congestion, inflammatory-cell infiltration, alveolar wall thickening, and lung parenchyma, compared with those from the control group (Fig. 2D, top-left). However, ZTOI appeared to offer excellent protection to lung tissues from RSV injury (Fig. 2D, bottom), which was further confirmed by the pathological scores from Table 1 (Fig. 2E). Additionally, the lung index of the lungs exposed to RSV was significantly increased compared with that of the control group, which was suppressed by the administration of ZTOI (Supporting information Fig. S3). Accumulating evidence suggested that ZTOI could effectively inhibit the inflammatory progression and pathological changes of the lungs infected by RSV.

### ZTOI inhibits RSV in young mice with RSV-induced pneumonia

**Fig. 3:**
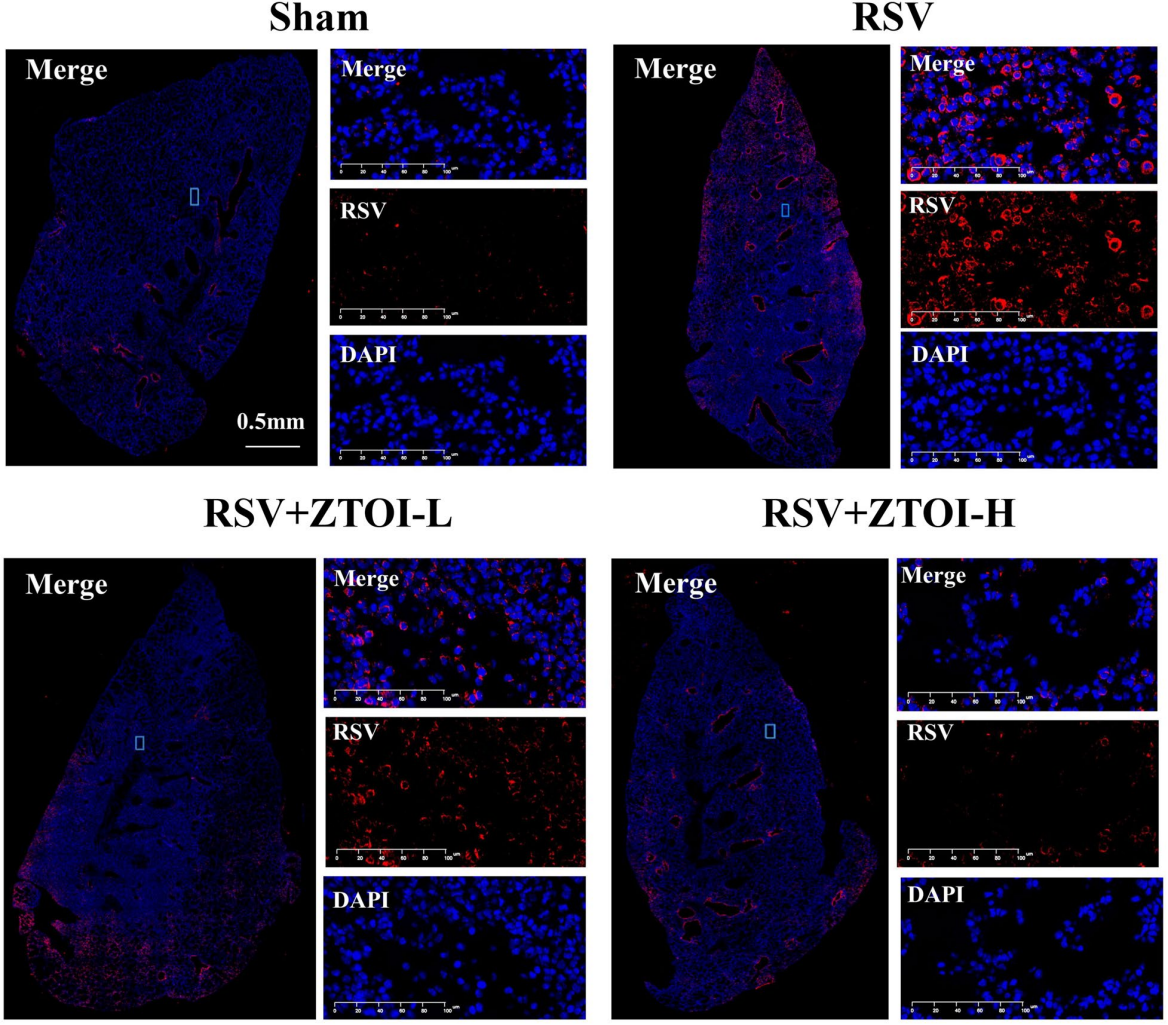
Representative immunofluorescence images stained with anti-RSV antibody (red) in lung tissues. The regions of interest (ROI) are boxed, and their magnified images are shown on the right

As anti-RSV antibodies can specifically bind to RSV-infected cells and tissues, or viral lysate, characterizing the content of RSV by IF, immunohistochemistry, and western blotting has widely been used in research related to RSV infection in recent years [32]. Therefore, we used IF with anti-RSV antibodies to evaluate the differences in RSV content between different groups. Compared with the control group, IF staining showed that the expression of RSV was substantially higher in the lung tissues of RSV mice, but was dose-dependently reversed by ZTOI treatment (Fig. 3 and Supporting information Fig. S4). The RNA expression levels of RSV in the lung tissues by real-time fluorescence quantitative PCR also indicated that ZTOI could inhibit RSV in young mice with RSV-induced lung inflammation (Supporting information Fig. S5).

### Quantitative measurement of multiple cytokines elucidates the potential mechanisms of ZTOI against RSV-induced lung inflammation

**Fig. 4:**
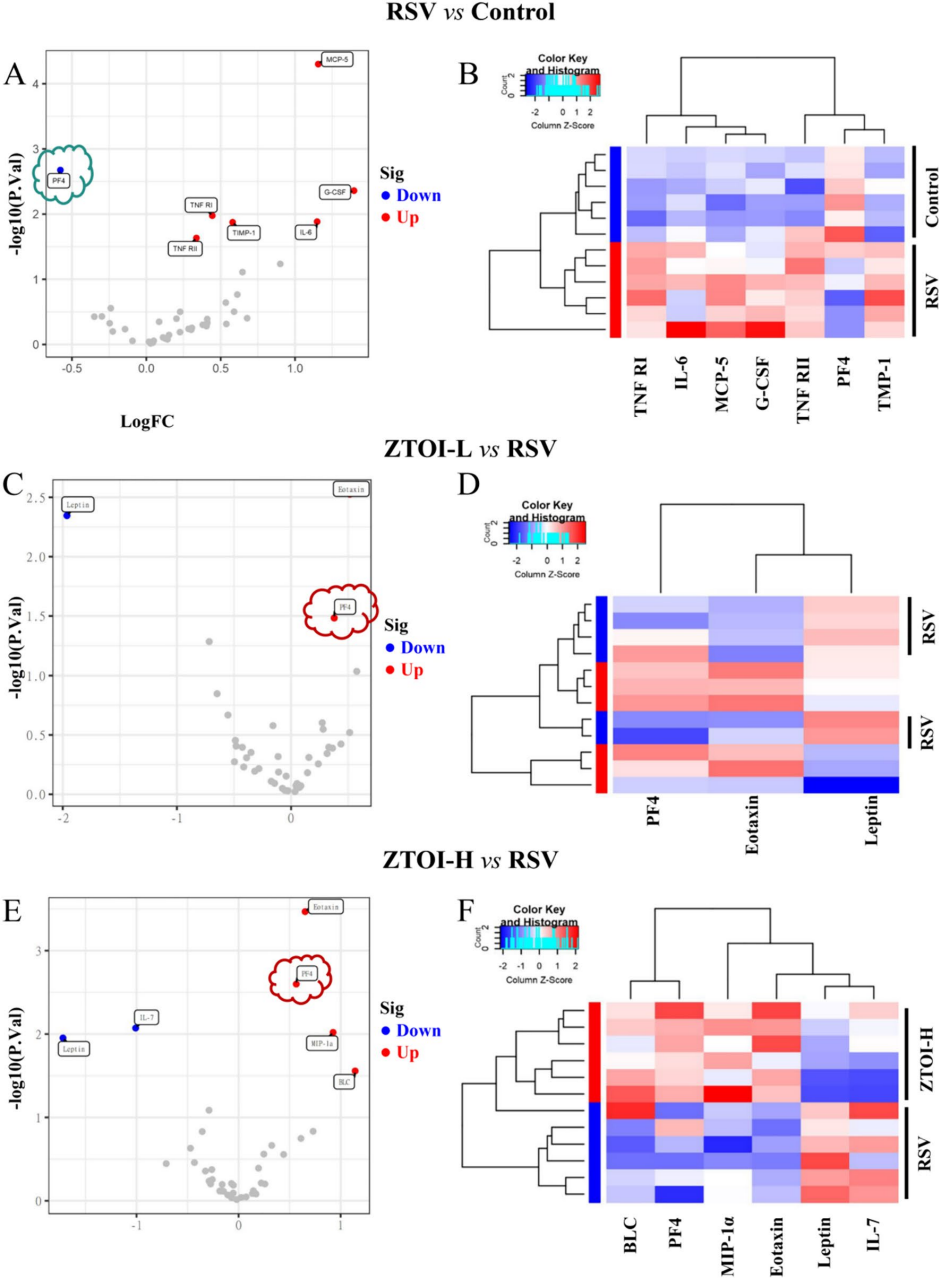
Volcano plot of 40 mouse cytokines (**A**, **C**, and **E**; red represents upward adjustment, blue represents downward adjustment, grey represents no significant difference) and heat map of differential mouse cytokines (**B**, **D**, and **F**; red series represent upward adjustment, and blue series represent downward adjustment)

The advantage of TCM in resisting viral pneumonia generally lies not in its killing of the virus, but in its regulation of inflammation [33]. A variety of cytokines play an important role in the occurrence and development of inflammation. Therefore, we examined inflammatory cytokines in the mouse lung tissues by mouse inflammation array, which contained 40 cytokines (Supporting Information Fig. S6). Compared with the control group, RSV infection upregulated the levels of tumor necrosis factor receptor type I (TNF-RI, also called p60), tumor necrosis factor receptor type II (TNF-R II), IL-6, monocyte chemotactic protein 5 (MCP-5), granulocyte colony-stimulating factor (G-CSF), and tropomyosin-1 (TMP-1), and downregulated the level of PF4, as shown in Fig. 4A and B, and Supporting information Table S1. Compared with the RSV group, the low-dose ZTOI treatment upregulated the levels of chemokines of PF4 and eotaxin, and downregulated the level of leptin, as shown in Fig. 4C and D, and Supporting information Table S2. The high-dose ZTOI treatment further upregulated the levels of chemokines of PF4, eotaxin, and newly added macrophage inflammatory protein 1 alpha (MIP-1α) and B-lymphocyte chemoattractant (BLC), and downregulated the levels of leptin and IL-7, as shown in Fig. 4E and F, and Supporting information Table S3. Among the significantly changed cytokines, the chemokine PF4 appeared particularly important and meaningful (Supporting Information Fig. S7). Infection with RSV significantly reduced PF4 content in the lung tissue of young mice. However, the ZTOI intervention reversed this phenomenon in a dose-dependent manner. Therefore, PF4 plays an important role in the anti–RSV-induced lung inflammation effect of ZTOI in young mice.

### PF4 is a key target for the treatment of RSV-induced pneumonia with ZTOI

**Fig. 5:**
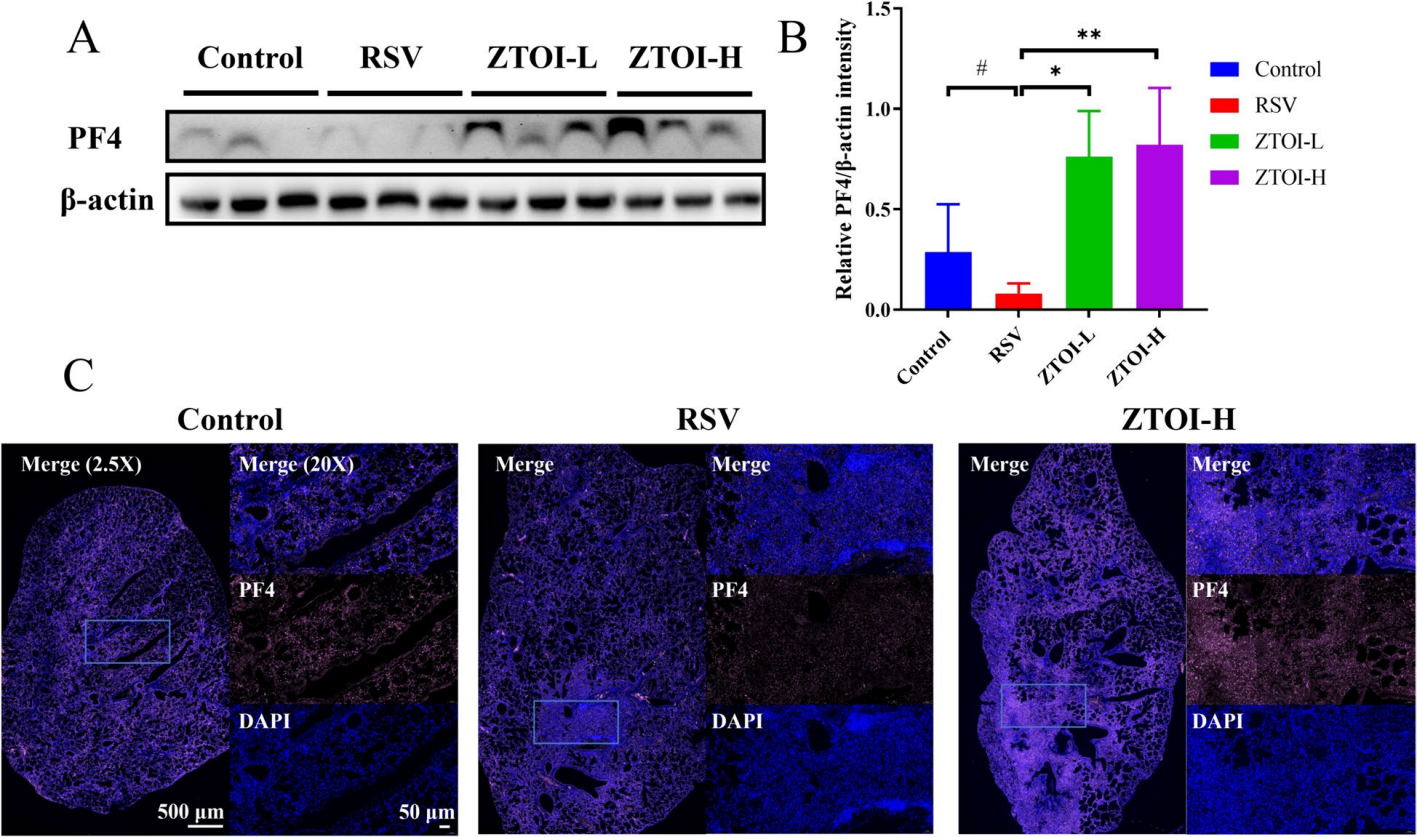
Western blot analysis of platelet factor 4 (PF4) in lung tissues of RSV-induced mice (**A**), densitometry analysis of PF4 expression of (**A**) (**B**, data are mean ± SED, n = 6, *P < 0.05 and **P < 0.01 *vs*. the RSV group, and #P < 0.05 *vs.* the Control group), and representative immunofluorescence images stained for PF4 (pink) in lung tissues (**C**, the regions of interest (ROI) are boxed, and their magnified images are shown on the right)

To further confirm the impact of RSV and ZTOI on PF4, western blotting, IF, and real-time fluorescence quantitative PCR were employed. The protein expression of PF4 was significantly downregulated by RSV and reversed by ZTOI in a dose-dependent manner (Fig. 5A and B). Consistent with the findings in western blotting, the lungs from the RSV-treated mice displayed weak IF responses of anti-PF4 antibody, while those IF responses were enhanced in the lungs from the ZTOI-treated mice (Fig. 5C). Although the gene expression of PF4 was also consistent with the above results, there was no significant difference (Supporting information Fig. S8). These results indicate that PF4 is a key target for the treatment of RSV-induced pneumonia with ZTOI.

### Curdione could be a main therapeutic material basis of ZTOI in target tissues

**Fig. 6:**
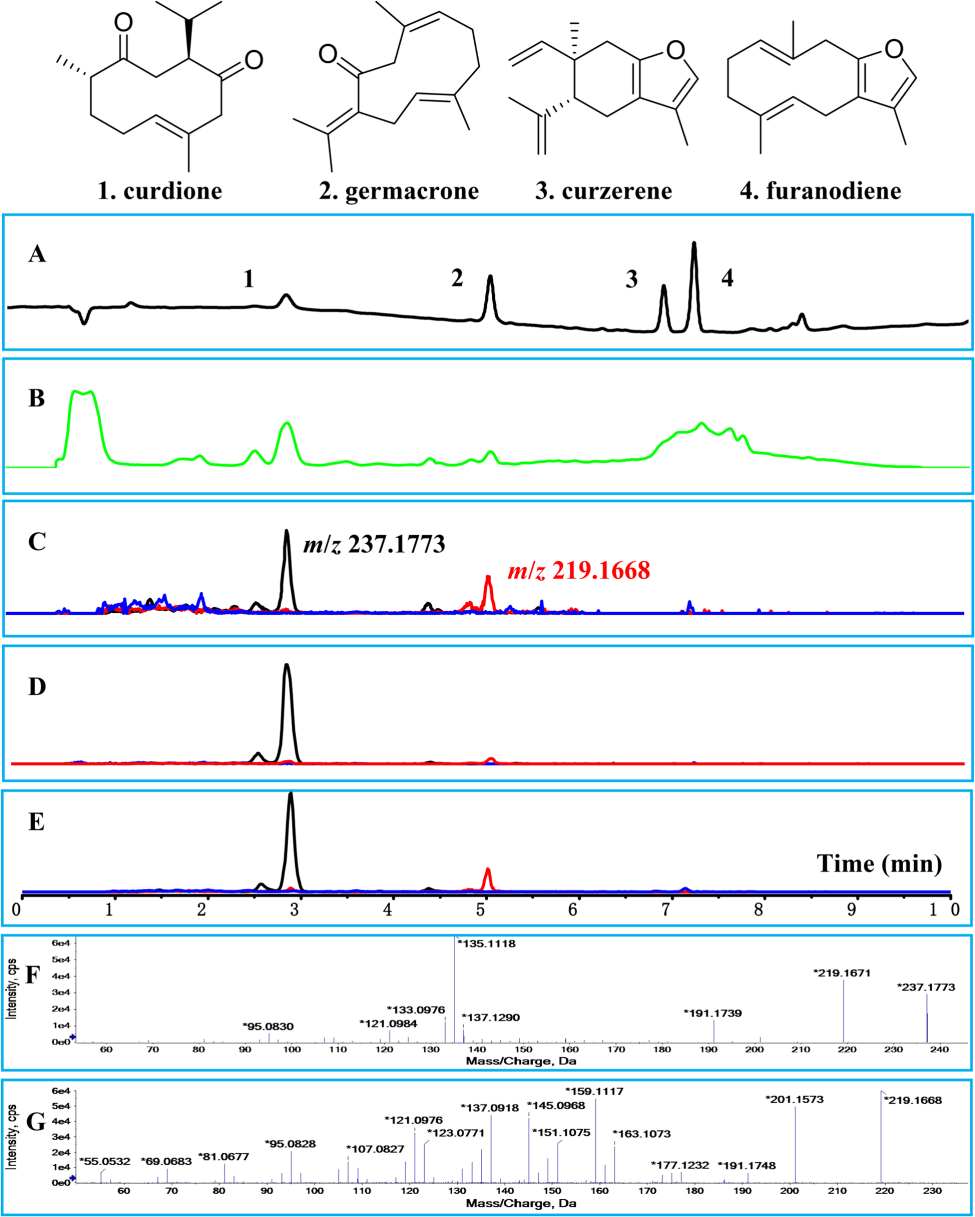
Screening of potential therapeutic material basis of ZTOI in target tissues. DAD chromatogram spectrum at 210 nm of ZTOI (**A**); total ion chromatogram spectrum of ZTOI (**B**); extracted ion chromatogram spectra of blood (**C**), feces (**D**), and lung (**E**) samples (black: *m*/*z* 237.1773 ± 0.005, red: *m*/*z* 219.1668 ± 0.005, blue: *m*/*z* 217.1538 ± 0.005); detailed fragmentation pathway of curdione (**F**) and germacrone (**G**)

To clarify the potential therapeutic material basis of ZTOI in lung and intestine tissues, we qualitatively monitored the chemical components of ZTOI in both lungs and intestines at different time points using non-targeted mass spectrometry. Previous studies have shown that the chemical compounds of ZTOI are rich in sesquiterpenes, mainly including curdione, furanodiene, germacrone, and curzerene (Fig. 6) [5, 6]. After intravenous injection of ZTOI, the prototype components of curdione were detected in the lung tissues and the feces collected from intestines. From the analysis results, sesquiterpenes, especially curdione, were the main enriched pharmacological substances in the lungs and intestines (Fig. 6A–E). The secondary mass spectra and fragmentation pathways of curdione and germacrone are shown in Fig. 6F and G and Supporting Information Fig. S9 and S10. Under the same sample-weight conditions, the peak areas of curdione in tissues extracted at different time points showed a downward trend as the time passed by (Supporting information Figures S11–S14). Therefore, sesquiterpenes, such as curdione and germacrone, can accumulate in both the lung and intestine, thereby exerting physiological functions.

### ZTOI regulates the gut microbiota of young mice

**Fig. 7:**
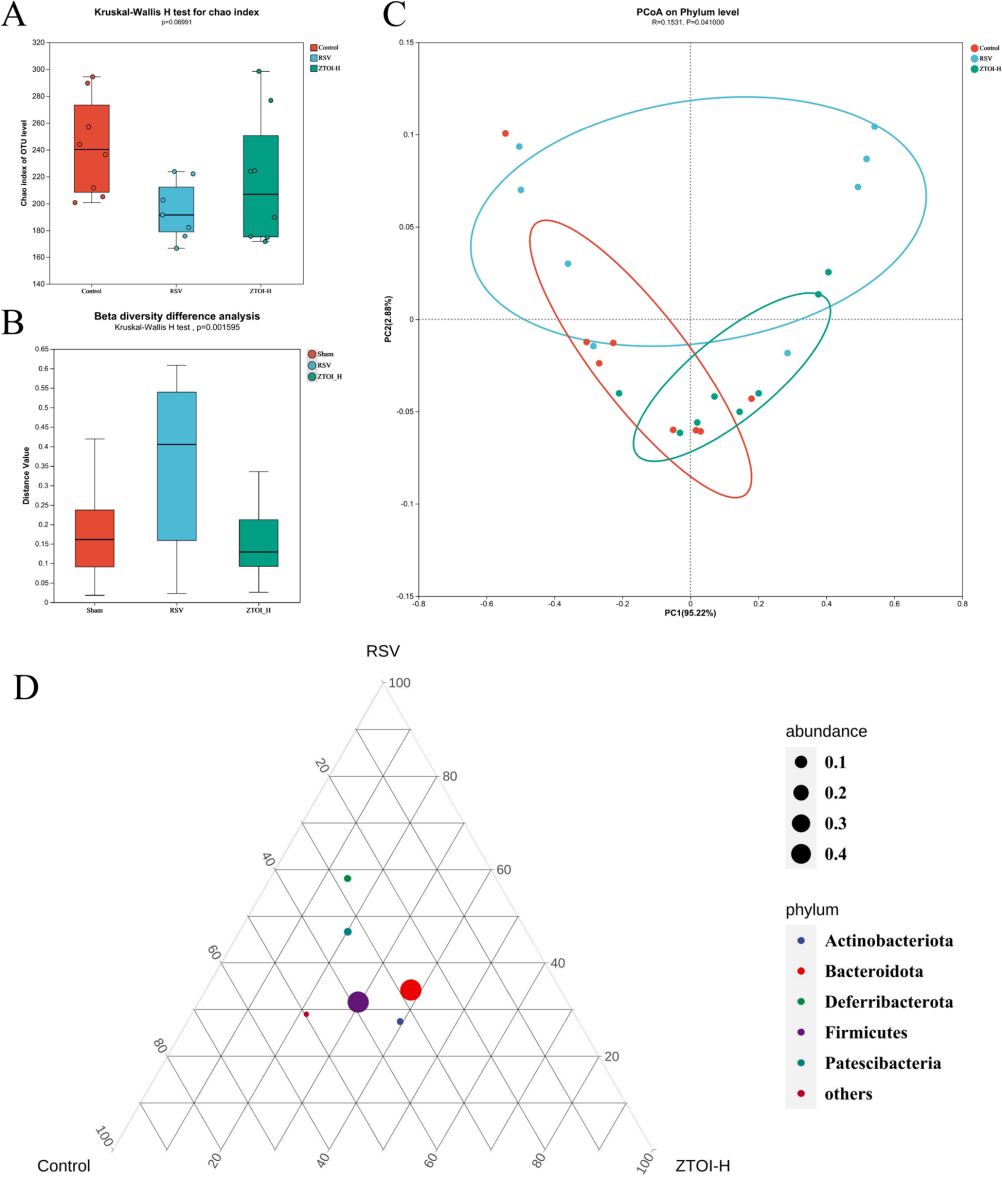
Analysis of alpha-diversity (**A**) and beta-diversity (**B**) of fecal microorganisms in three groups of young mice based on OTU levels; PCoA based on the phylum level (**C**, each point represents a sample and the distance between points is used to map the size of the difference in the structural composition of the gut flora between the two samples); and ternary analysis of the fecal microorganisms composition in the control, RSV, and ZTOI-H groups at the phylum level (**D**)

The changes in gut microbiota play an important regulatory role in the occurrence and development of diseases. Due to the fragility of the gut microbiota in children, we investigated the effect of ZTOI on the gut microbiota of young mice. After confirming that the pharmacological substances can reach the intestine, we used 16S rRNA-sequencing technology to study the effect of ZTOI on the intestinal bacteria. In all young mice fecal samples, we identified 1080128 16S rRNA gene sequences, all of which were classified at 97% similarity according to OTU and subsequent bioinformatics. In three groups, 414 OTUs belonging to 9 phyla, 13 classes, 39 orders, 63 families, 120 genera, and 169 species were identified. The curves in the Shannon plots (Supporting information Fig. S15) all converged to parallel straight lines, indicating that the richness and evenness of bacterial community in the samples were sufficient. The Chao values in the RSV and ZTOI-H groups were lower than that in the control group (*P* = 0.0699), indicating that the abundance of intestinal flora was reduced by RSV and slightly reversed by ZTOI (Fig. 7A). For beta diversity, Kruskal–Wallis H test (Fig. 7B) and principal co-ordinates analysis (PCoA, Fig. 7C) were applied to compare the structure of the microbial community in diverse groups. RSV infection caused disruption of the gut microbiota in young mice. The intervention with ZTOI was not able to reverse the disruption of the intestinal flora caused by RSV, but changed the composition of the intestinal flora, mainly including Patescibacteria and Deferribacterota phyla (Fig. 7D). At the genus level, the significantly affected bacterial genera included Rikenellaceae_rc9_gut_group, Alloprevotella, Mucispirillun, Lachnospiraceae_UCG-001, Roseburia, Candidatus_Saccharimonas, and Norank_f_Lachnospiraceae (Supporting information Fig. S16). However, the roles of these bacteria in RSV-induced lung inflammation still need further clarification.

## Discussion

Discovering effective drugs to combat RSV has always been challenging. Ethnic drugs derived from plants have long played an important role in combating diseases. A series of TCM or natural drugs against viral pneumonia have been reported in recent years [34, 35]. ZTOI, composed of essential oil (typically called ZTO in TCM) isolated from herbal medicine *Curcuma wenyujin* Y. H. Chenet C. Ling, has widely been used against pneumonia caused by multiple agents in clinical practice. For RSV-induced lung inflammation, given the frequent combination of TCM and western medicines in clinical practice, only one clinical experiment has shown that the single use of ZTO could improve clinical symptoms and shorten the course of the disease [36]. In vitro cell models have indicated that ZTO can significantly inhibit RSV-infected cells [37]. Reported in vivo animal experiments have only focused on the histopathological changes [37, 38], which seriously limited the clinical application of ZTO. Therefore, systematic and multi-indicator research on the efficacy of ZTO against RSV-induced pneumonia is needed to support its in-depth development. From our experimental results, it is apparent that ZTOI not only improves pathological damage of the lungs caused by RSV, but also reduces inflammatory markers such as MPO, SAA, CRP, TLSP, IL-1β, and IL-6, thereby inhibiting the occurrence and development of RSV-induced pneumonia.

Mouse inflammation array evaluation further elucidated the role of cytokines in RSV infection and ZTOI treatment. The difference in serum cytokines between the RSV group and the control group indicated that RSV was able to significantly upregulate MCP-5, G-CSF, IL-6, TIMP-1, TNF-RI, and TNF-RII, and downregulate PF4, as shown in Fig. 4A and B, and Supporting Information Table S1. The Kyoto Encyclopedia of Genes and Genomes (KEGG) enrichment on these proteins (Supporting information Fig. S17D) showed that the different cytokines were dramatically enriched in the viral protein interaction with cytokine and the cytokine receptor pathway (Supporting information Fig. S18) and the TNF signaling pathway (Supporting information Fig. S19 and S20). It has been shown that replication-defective viral genome (DVG) of RSV is its primary trigger of antiviral immunity during replication, and can also facilitate viral persistence. This pro-survival effect is dependent on the activity of the TNF-RII/TRAF1 pathway [39]. Therefore, the TNF signaling pathway plays an essential role in RSV-induced pulmonary inflammation [40]. However, compared with the RSV group, ZTOI treatment significantly upregulated the levels of chemokines of PF4, eotaxin, MIP-1α, and BLC, and downregulated the levels of leptin and IL-7 (Supporting information Fig. S21). KEGG enrichment on these proteins (Supporting Information Fig. S22D) showed that the different cytokines were dramatically enriched in the viral protein interaction with cytokine–cytokine receptor interaction (Supporting Information Fig. S23) and chemokine signaling pathway (Supporting Information Fig. S24). Leptin, one of the adipokines with proinflammatory effect, shows high levels in infant serum and is associated with a greater frequency of viral coinfection and low RSV viral load in infants [41]. IL-7, a cytokine for T-cell development as well as for the survival and homeostasis of mature T cells, has shown a persistent nasal hypersecretion in RSV-infected children [42]. The inhibitory effect of ZTOI on leptin and IL-7 may be beneficial for the prognosis of RSV infection. Additionally, the high levels of chemokines such as eotaxin, MIP-1α, and BLC indicated that the ZTOI-treated groups still suffered from inflammatory reactions, which might be caused by the severe RSV infection twice within 5 days. However, there was no significant pathological damage in the lung tissues of the ZTOI-L and ZTOI-H groups (Fig. 2D), indicating that ZTOI could avoid triggering an inflammatory overreaction.

PF4 is also known as CXCL4: it is the first discovered chemokine and is mainly produced by platelets. In recent years, it has been shown that PF4 can also be produced in macrophages and activated T cells. PF4 not only shows extensive regulatory effects on inflammation [43], but has also been identified as a strong and effective RSV inhibitor by directly blocking the attachment of RSV virus particles to heparin sulfate receptor [44]. Contrary to our research findings, previous studies have shown elevated PF4 concentrations in both plasma and alveolar lavage fluid samples of RSV-infected mice and patients [44]. It should be noted that PF4 gene knockout can lead to reduced clearance of influenza A virus, significant infiltration of neutrophils into the lungs, and lung inflammation during early infection. However, in the late stage of infection, compared with wild-type mice, leukocytes are extensively aggregated and lung tissue pathology is more severe in the PF4 gene knockout mice. Therefore, the lack of PF4 might inhibit the recruitment of neutrophils into the lungs of infected mice [45]. The conflicting therapeutic effects of PF4 also occur in liver injury [46, 47]. Therefore, PF4 may have completely opposite regulatory patterns in different organs and physiological environments, thereby promoting or inhibiting the development of inflammation.

**Fig. 8:**
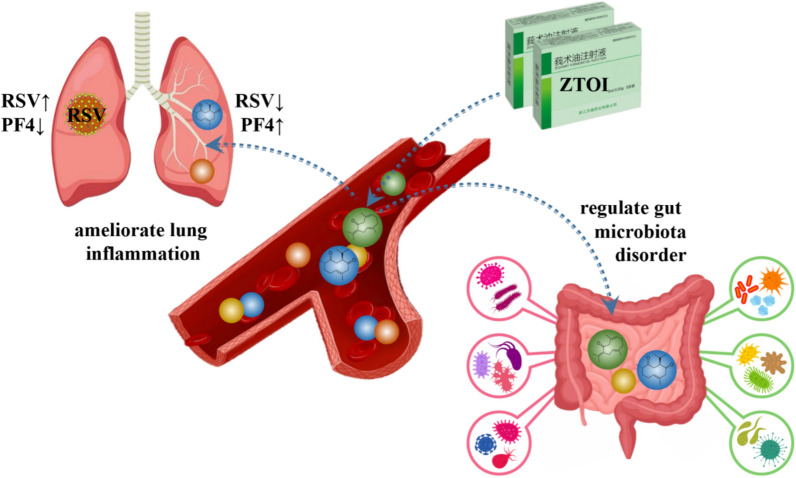
The proposed underlying mechanism of ZTOI against lung inflammation and gut microbiota disorder in respiratory syncytial virus–infected young mice

The role of the lungs in the hematopoietic system is underestimated. It has been shown that the lungs are responsible for producing more than half of the platelets in a mouse model [48]. Simultaneously, platelets not only directly participate in immune defense, but also assist and regulate innate immune cells [49]. In addition, the in vivo and in vitro experimental results showed that oral administration of curdione, one of the main compounds from ZTOI/ZTO, could be detected both in the serum and platelet lysate of mice and patients. Our research also indicates that curdione can be enriched in target organs (Fig. 8). Therefore, the regulatory effect of curdione on platelets deserves in-depth research.

The gut microbial population is very stable in terms of structural composition, but it is also susceptible to modification by various factors, such as age, stress, and antibiotic use [50–53]. Intestinal flora can directly alter pulmonary immunity [54]. Infants exhibit a rapid increase in intestinal flora diversity by 1 year of age. The composition of the intestinal flora becomes more stable from 1 to 5 years of age, but the rate of growth of flora diversity also decreases significantly. However, they have lower intestinal flora in terms of numbers and species compared with adults [55], so it is easy to cause an imbalance of intestinal flora. Therefore, we should focus on the treatment of gut microbiota in infants with RSV infection.

## Conclusion

With our in vivo demonstration of the anti–RSV-induced lung inflammation effect of ZTOI, we found that ZTOI could restrict the inflammatory reaction and inhibit virus replication. Notably, RSV intervention downregulated PF4 and resulted in severe lung damage. However, the intervention with ZTOI reversed the decrease in PF4 and repaired the damage of lung tissue. Therefore, ZTOI can protect young mice against RSV-induced lung inflammation via PF4. Our study also indicated that sesquiterpenes, especially curdione, can be enriched in the lungs and intestines, thereby exerting its anti-inflammatory and regulatory effects on gut microbiota. Therefore, ZTOI exerts salutary effects on RSV-induced lung inflammation and may serve as a candidate therapy in clinical practice.

### Supplementary Information

Supplementary Material 1.

## Data Availability

The data that support the findings of this study are available from the corresponding author upon reasonable request.

## Abbreviations

BLC: B-lymphocyte chemoattractant
CRP: C-reactive protein
HE: Hematoxylin and eosin
KEGG: Kyoto encyclopedia of genes and genomes
LMICs: Low-income and middle-income countries
MIP-1α: Macrophage inflammatory protein 1 alpha
MPO: Myeloperoxidase
PF4: Platelet factor 4
RSV: Respiratory syncytial virus
SAA: Serum amyloid A
TCM: Traditional Chinese medicine
TSLP: Thymic stromal lymphoprotein
ZTO: Zedoary turmeric oil
ZTOI: Zedoary turmeric oil injection
