# Supplementary material for: Zedoary turmeric oil injection ameliorates lung inflammation via platelet factor 4 and regulates gut microbiota disorder in respiratory syncytial virus-infected young mice
Source: Chin Med. 2024 Jun 11;19:83. doi: 10.1186/s13020-024-00954-6 (PMC11165741; doi:10.1186/s13020-024-00954-6)
Supplement: Supplementary file 1 — Supplementary Material 1. [file 13020_2024_954_MOESM1_ESM.pdf]

Zedoary turmeric oil injection ameliorates lung inflammation *via* platelet factor 4 and regulates gut microbiota disorder in respiratory syncytial virus–infected young mice

Yu-Zhuo Wu[1, #], Qian Zhang[1, #], Hua Li [1, 3], Cheng-Xi Jiang[2], Xiao-Kun Li[2], Hong-Cai Shang[1, *], Sheng Lin[1, *]

[1]Key Laboratory of Chinese Internal Medicine of Ministry of Education and Beijing, Dongzhimen Hospital, Beijing University of Chinese Medicine, Beijing 100700, China

[2]School of Pharmacy, Wenzhou Medical University, Wenzhou 325035, China

[3]Wuya college of innovation, Key Laboratory of Structure-Based Drug Design & Discovery, Ministry of Education, Shenyang Pharmaceutical University, Shenyang 110016, China

SUPPORTING INFORMATION

[#]These authors contributed equally to this work.
[*]Corresponding Author
Hongcai Shang, Ph.D.
Professor
Key Laboratory of Chinese Internal Medicine of Ministry of Education and Beijing, Dongzhimen Hospital, Beijing University of Chinese Medicine
Tel: +86-010-84013404, Email: shanghongcai@126.com

Sheng Lin, Ph.D.
Professor
Key Laboratory of Chinese Internal Medicine of Ministry of Education and Beijing, Dongzhimen Hospital, Beijing University of Chinese Medicine
Tel: +86-010-84013404, Email: lsznn@bucm.edu.cn

CONTENTS

Table S1 Differential cytokines in the RSV group compared to the control group 3
Table S2 Differential cytokines in the ZTOI-L group compared to the RSV group 3
Table S3 Differential cytokines in the ZTOI-H group compared to the RSV group 3
Figure S1 Representative histological images of hematoxylin and eosin–stained lung section.
Figure S2 Changes of key inflammatory indicators in lung and serum. 4
Figure S3 Lung indexes evaluation. 4
Figure S4 Densitometry analysis of RSV expression of Figure 3. 4
Figure S5 RNA expression level of RSV 5
Figure S6 Forty cytokines and their distribution in chip. 5
Figure S7 Forty cytokines and their distribution in chip. 6
Figure S8 RNA expression level of PF4. 6
Figure S9 Detail fragmentation pathway of curdione. 6
Figure S10 Detail fragmentation pathway of germacrone. 6
Figure S11 Extracted ion chromatogarm spectra of blood samples 7
Figure S12 Extracted ion chromatogarm spectra of faeces 7
Figure S13 Extracted ion chromatogarm spectra of lung 8
Figure S14 Extracted ion chromatogarm spectra of blank 8
Figure S15 Shannon plots. 9
Figure S16 Ternary analysis of the fecal microorganisms composition in the control, RSV and ZTOI-H groups at genus level. 9
Figure S17 Differential cytokines analysis of the RSV group compared to the control group 10
Figure S18 Viral protein interaction with cytokine and cytokine receptor pathway. 10
Figure S19 TNF signaling pathway. 11
Figure S20 Relationship of differential cytokines in the RSV group compared to the control group.11
Figure S21 Relationship of differential cytokines in the ZTOI group compared to the RSV group12
Figure S22 Differential cytokines analysis of the ZTOI-H group compared to the RSV group. 12
Figure S23 Cytokine-cytokine receptor interaction. 13
Figure S24 Chemokine signaling pathway. 13
Figure S25 Original western blot figures of figure 5A. 14

Table S1 Differential cytokines in the RSV group compared to the control group

proteinID	AveExp.RSV	AveExp.control	logFC	P.Value	adj.P.Val	foldchange	Regulation
MCP-5	5.849528824	4.693156017	1.156372806	4.99E-05	0.001997274	2.228963213	up
PF4	9.834934408	10.41252963	-0.577595221	0.00210932	0.042186402	0.67007978	down
G-CSF	5.97070986	4.574167595	1.396542265	0.004361577	0.058154359	2.632698415	up
IL-6	6.474537973	5.325964586	1.148573388	0.013050003	0.088826622	2.216945628	up
TIMP-1	10.87994759	10.29934736	0.580600233	0.013323993	0.088826622	1.495471309	up
TNF RI	6.962431912	6.518747816	0.443684096	0.010555925	0.088826622	1.360073008	up
TNF RII	9.01320189	8.676150646	0.337051244	0.023268605	0.132963456	1.263172129	up

Table S2 Differential cytokines in the ZTOI-L group compared to the RSV group

proteinID	AveExp.ZTOI-L	AveExp.RSV	logFC	P.Value	adj.P.Val	foldchange	Regulation
Eotaxin	6.125430607	5.610768054	0.514662553	0.002976884	0.09015212	1.428659936	up
Leptin	5.839036866	7.802353049	-1.963316183	0.004507606	0.09015212	0.256438329	down
PF4	10.21286212	9.834934408	0.377927712	0.032887245	0.438496595	1.299473949	up

Table S3 Differential cytokines in the ZTOI-H group compared to the RSV group

proteinID	AveExp.ZTOI-H	AveExp.RSV	logFC	P.Value	adj.P.Val	foldchange	Regulation
Eotaxin	6.263006278	5.610768054	0.652238224	0.000340367	0.013614665	1.571604522	up
PF4	10.40039339	9.834934408	0.565458982	0.00251423	0.050284596	1.47985824	up
IL-7	6.899453121	7.908011016	-1.008557894	0.008483019	0.089127715	0.497042839	down
Leptin	6.081738435	7.802353049	-1.720614614	0.011140964	0.089127715	0.303419431	down
MIP-1a	4.087712069	3.161310814	0.926401255	0.009548996	0.089127715	1.900529284	up
BLC	10.2459607	9.103534356	1.142426341	0.027715165	0.184767769	2.207519743	up

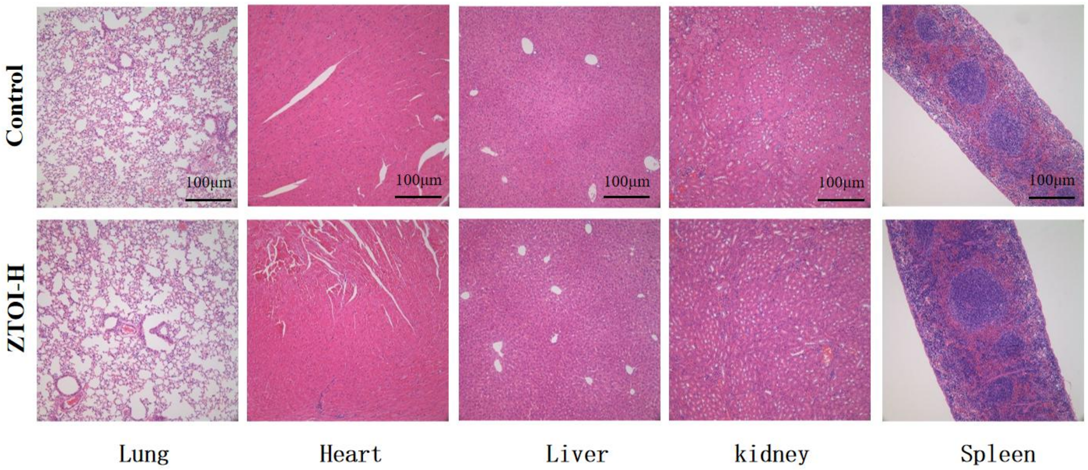

Figure S1 Representative histological images of hematoxylin and eosin–stained lung section (n=5).

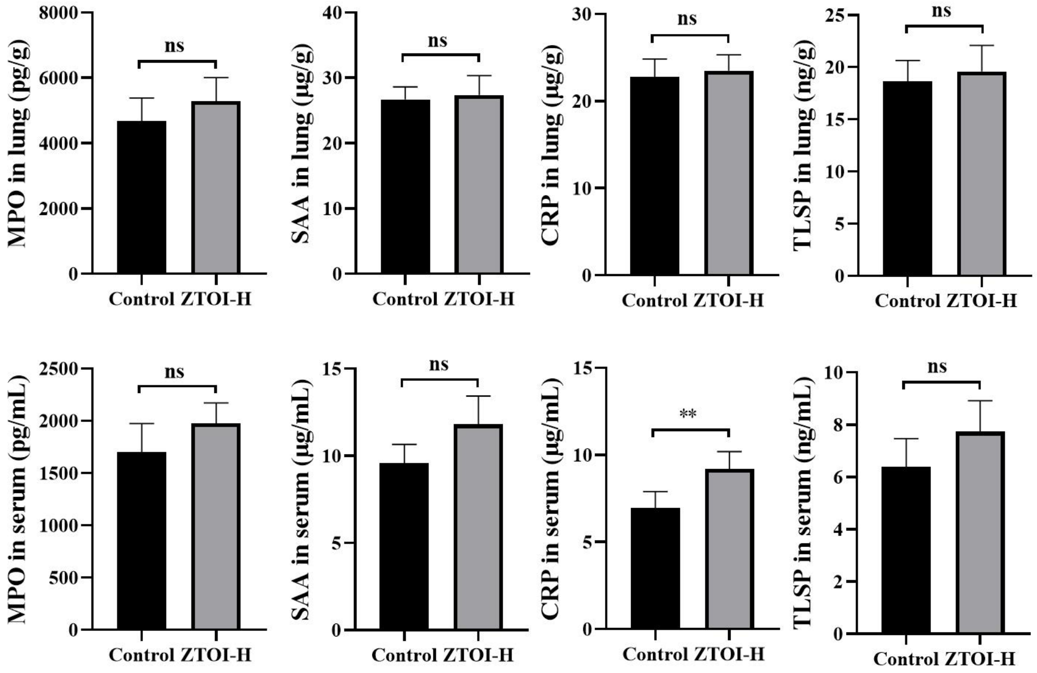

Figure S2 Changes of key inflammatory indicators in lung and serum (myeloperoxidase, MPO; serum amyloid A, SAA; C-reactive protein, CRP; thymic stromal lymphoprotein, TSLP; n = 6; data are presented as Means ± SD; ** P < 0.01, compared with the control group.

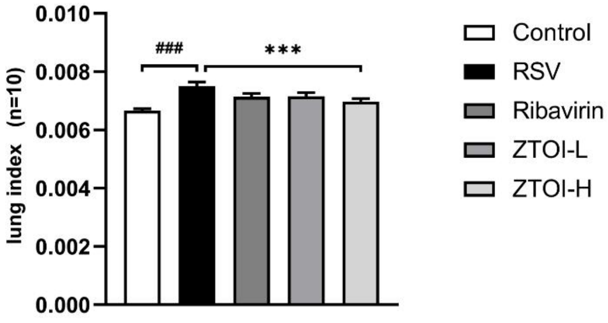

Figure S3 Lung indexes evaluation.

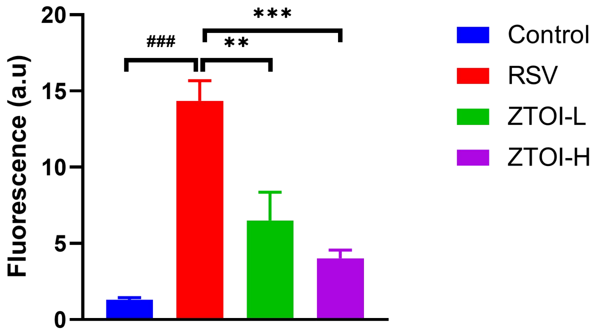

Figure S4 Densitometry analysis of RSV expression of Figure 3 (n=3; ** P < 0.01, *** P < 0.001, compared with RSV group; ### P < 0.001, compared with control group; data presented as means ± standard error of mean).

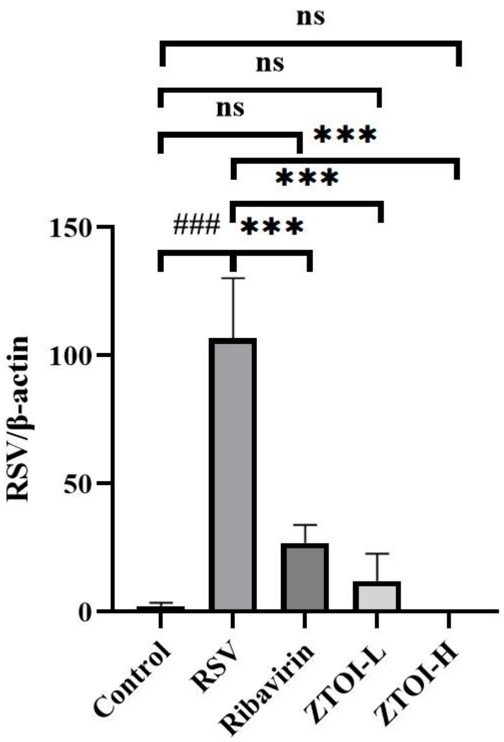

Figure S5 RNA expression level of RSV, n > 6; *** P < 0.001, compared with the RSV group; ### *P* < 0.001, compared with the control group; data presented as mean ± standard error of mean)

Each antibody is printed in quadruplicate horizontally			
	1 2 3 4	1 2 3 4	1 2 3 4
A	POS1	POS2	BLC (CXCL13)
B	CD30 Ligand	Eotaxin-1 (CCL11)	Eotaxin-2 (MPIF-2)
C	Fas Ligand	GCSF	GM-CSF
D	ICAM-1 (CD54)	IFN-gamma	IL-1 alpha
E	IL-1 beta	IL-2	IL-3
F	IL-4	IL-5	IL-6
G	IL-7	IL-10	IL-12 p70
H	IL-13	IL-15	IL-17A
I	IL-21	KC (CXCL1)	Leptin
J	LIX	MCP-1 (CCL2)	MCP-5
K	M-CSF	MIG (CXCL9)	MIP-1 alpha (CCL3)
L	MIP-1 gamma	Platelet Factor 4	RANTES (CCL5)
M	TARC (CCL17)	I-309 (TCA-3)	TIMP-1
N	TNF-alpha	TNF RI	TNF RII

Figure S6 Forty cytokines and their distribution in chip.

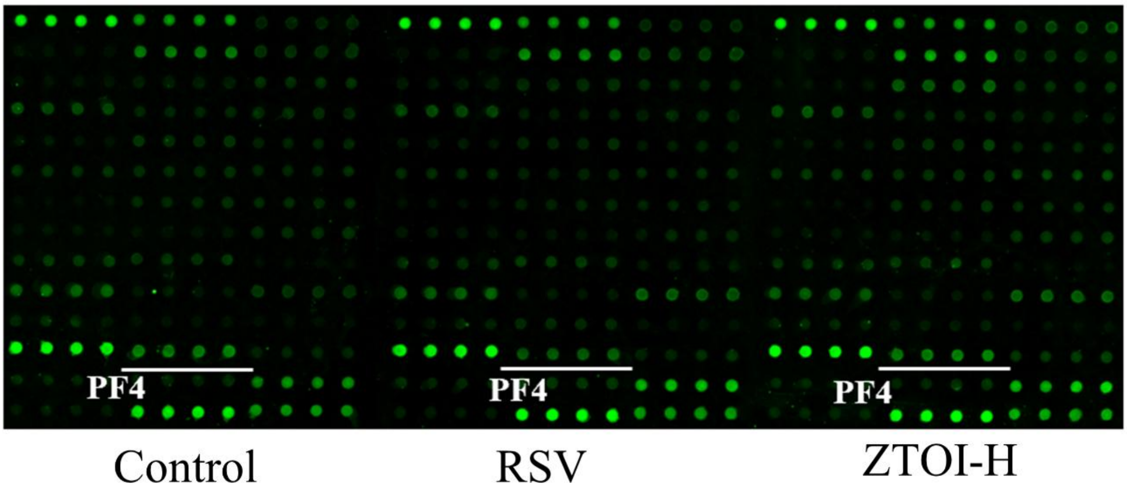

Figure S7 Forty cytokines and their distribution in chip.

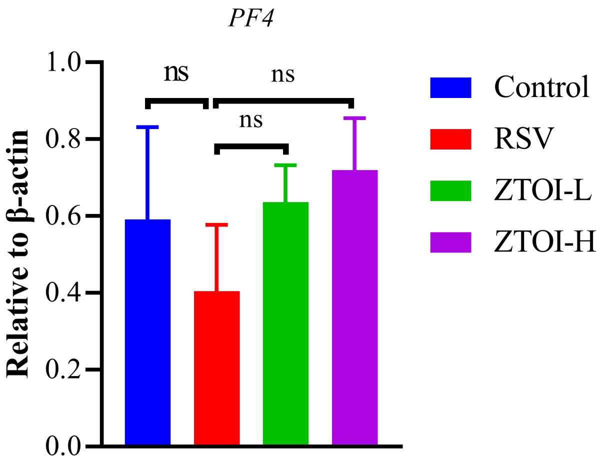

Figure S8 RNA expression level of PF4.

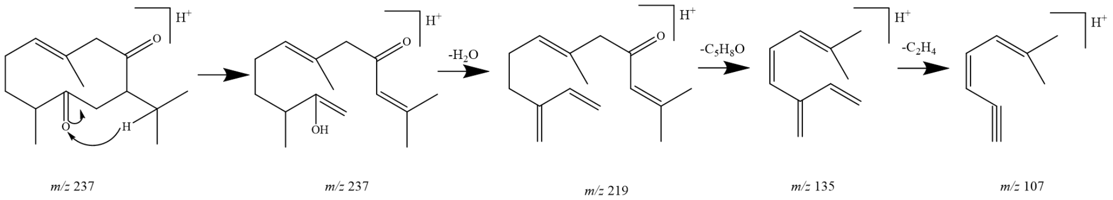

Figure S9 Detail fragmentation pathway of curdione.

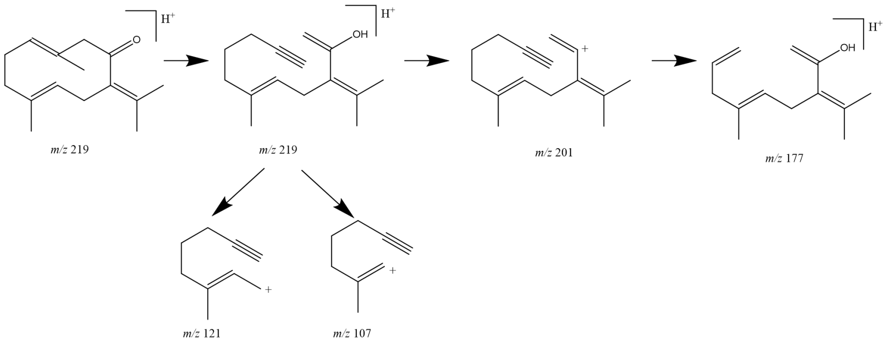

Figure S10 Detail fragmentation pathway of germacrone.

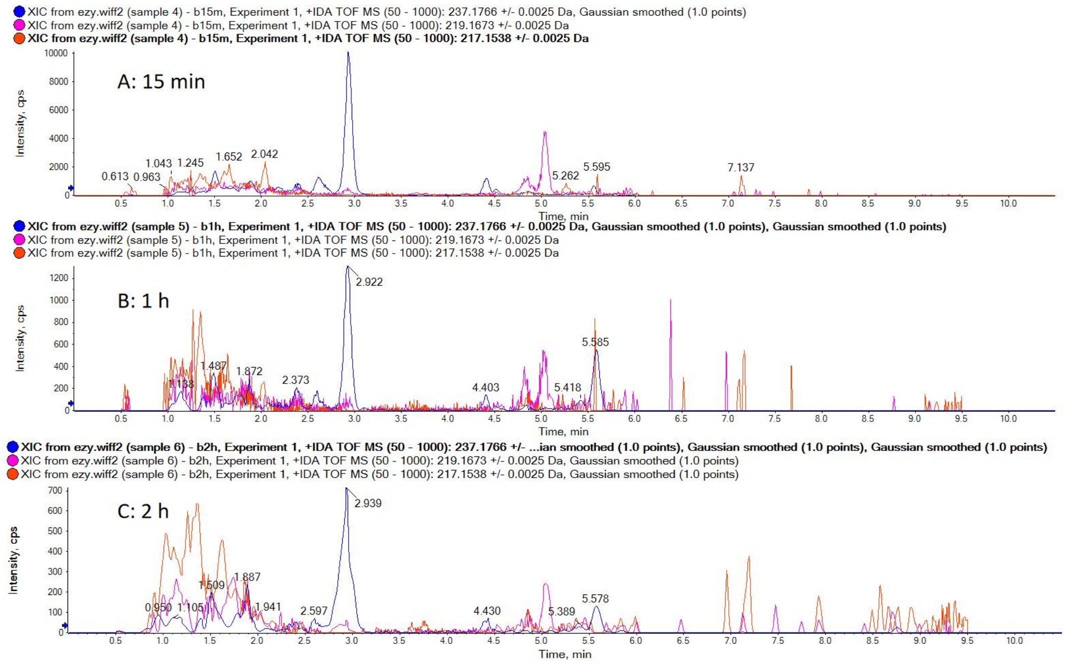

Figure S11 Extracted ion chromatogarm spectra of blood samples (blue: m/z 237.1773±0.005, red: m/z 219.1668±0.005, orange: m/z 217.1538±0.005)

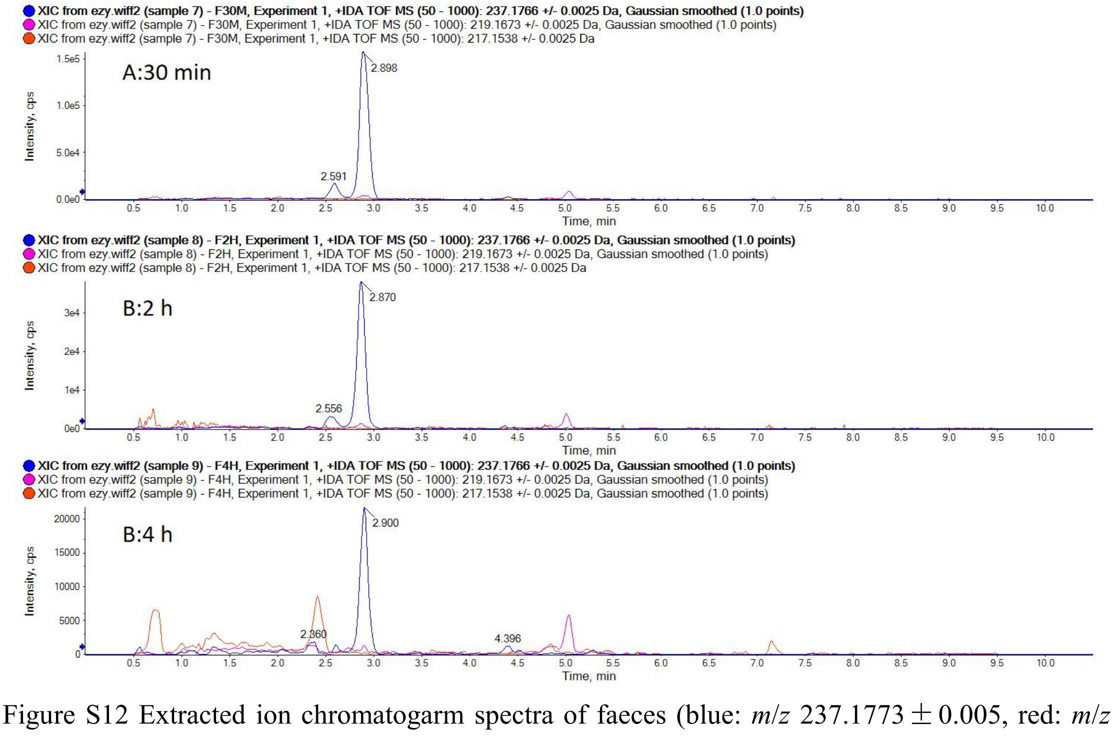

Figure S12 Extracted ion chromatogarm spectra of faeces (blue: m/z 237.1773±0.005, red: m/z 219.1668±0.005, orange: m/z 217.1538±0.005)

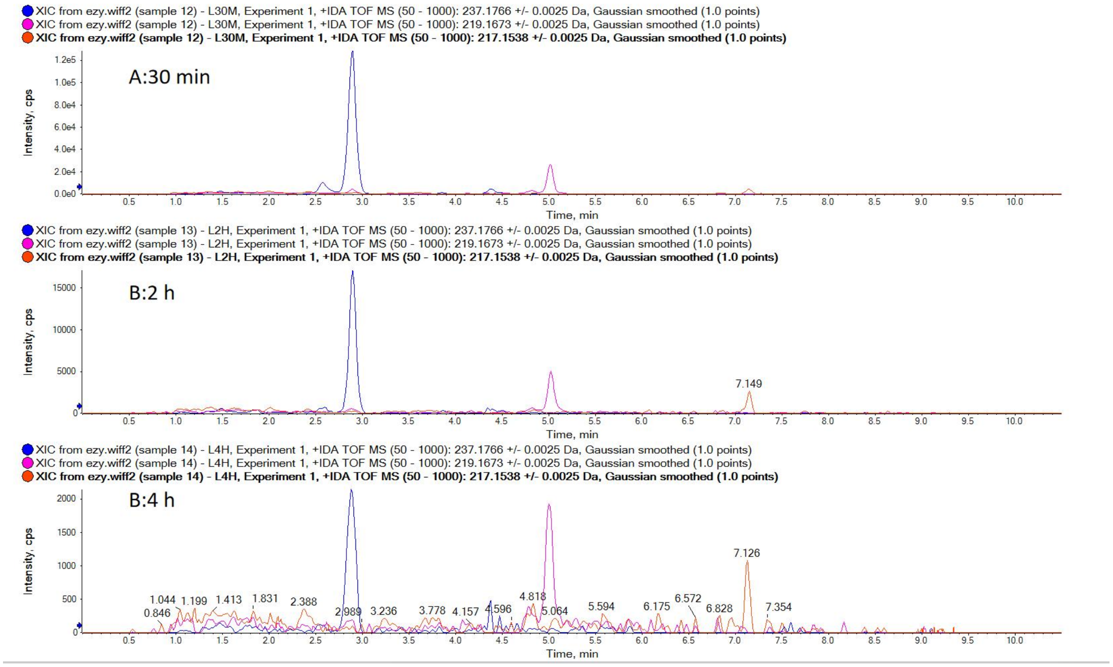

Figure S13 Extracted ion chromatogarm spectra of lung (blue: *m*/*z* 237.1773 ± 0.005, red: *m*/*z* 219.1668 ± 0.005, orange: *m*/*z* 217.1538 ± 0.005)

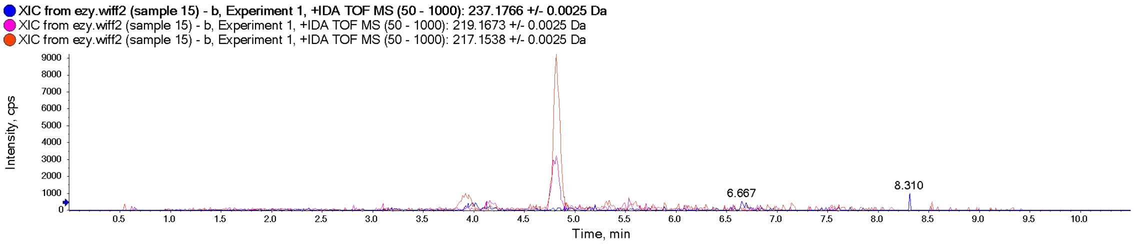

Figure S14 Extracted ion chromatogarm spectra of blank (blue: *m*/*z* 237.1773 ± 0.005, red: *m*/*z* 219.1668 ± 0.005, orange: *m*/*z* 217.1538 ± 0.005)

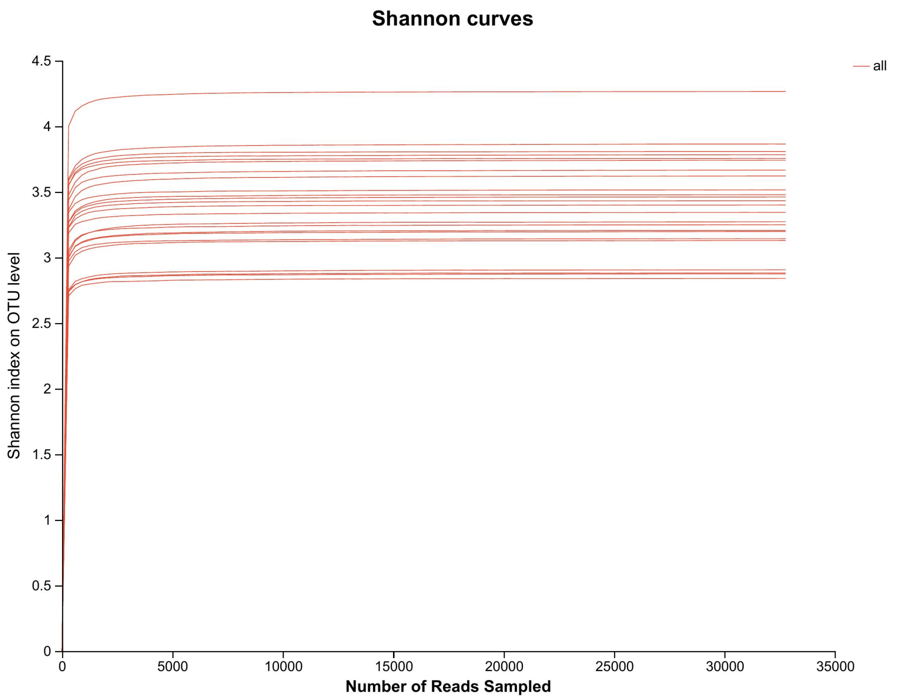

Figure S15 Shannon plots.

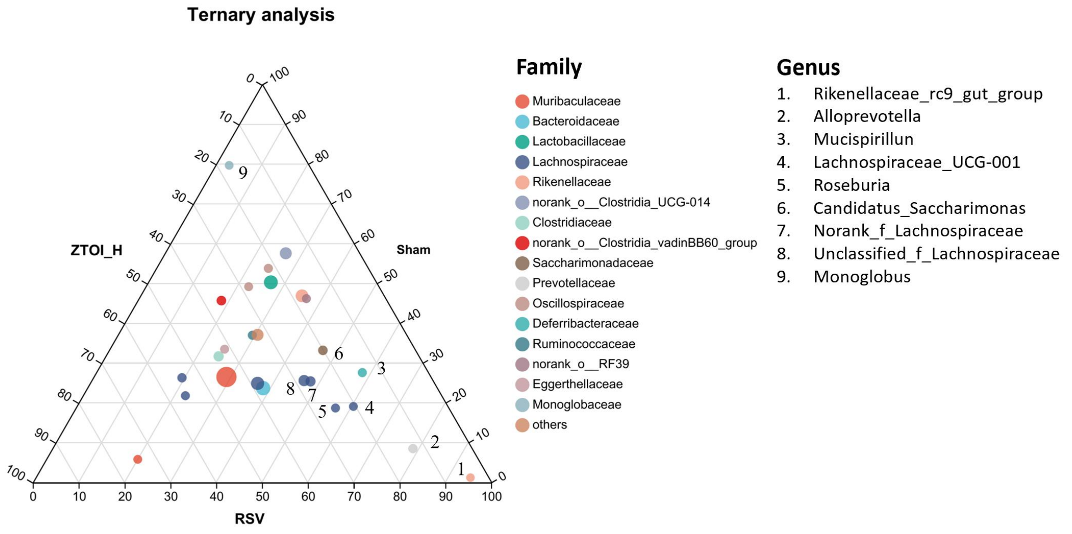

Figure S16 Ternary analysis of the fecal microorganisms composition in the control, RSV and ZTOI-H groups at genus level.

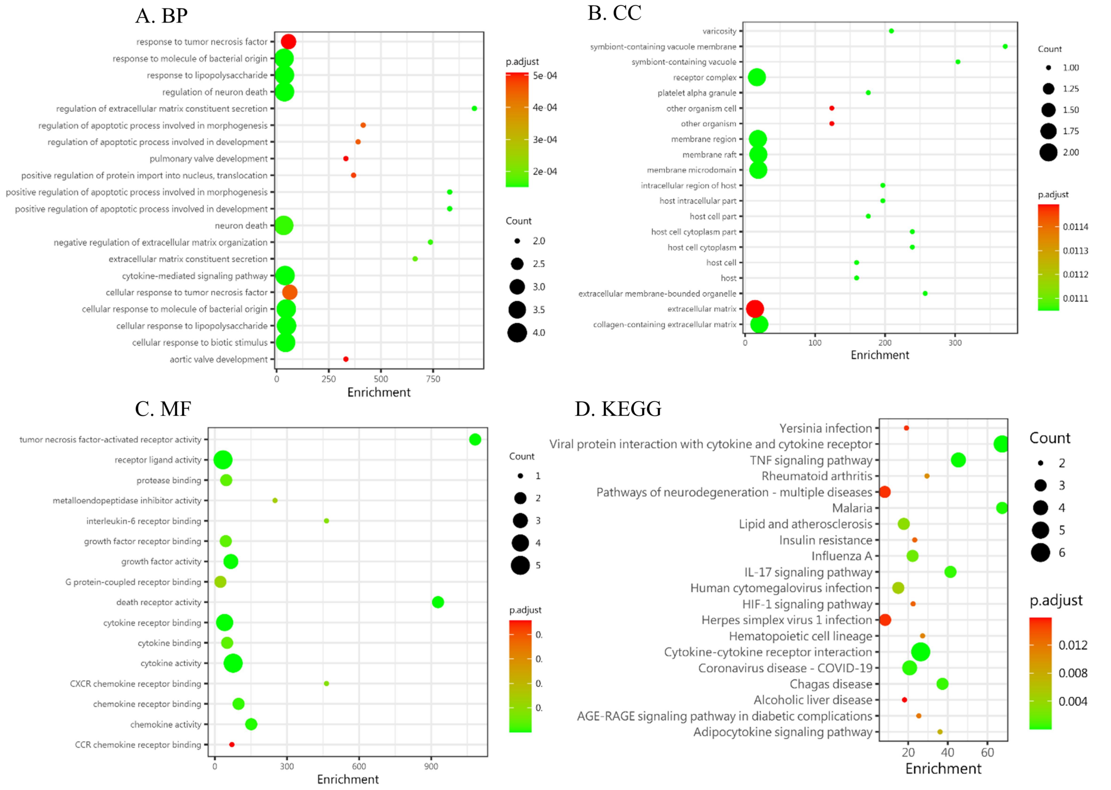

Figure S17 Differential cytokines analysis of the RSV group compared to the control group .

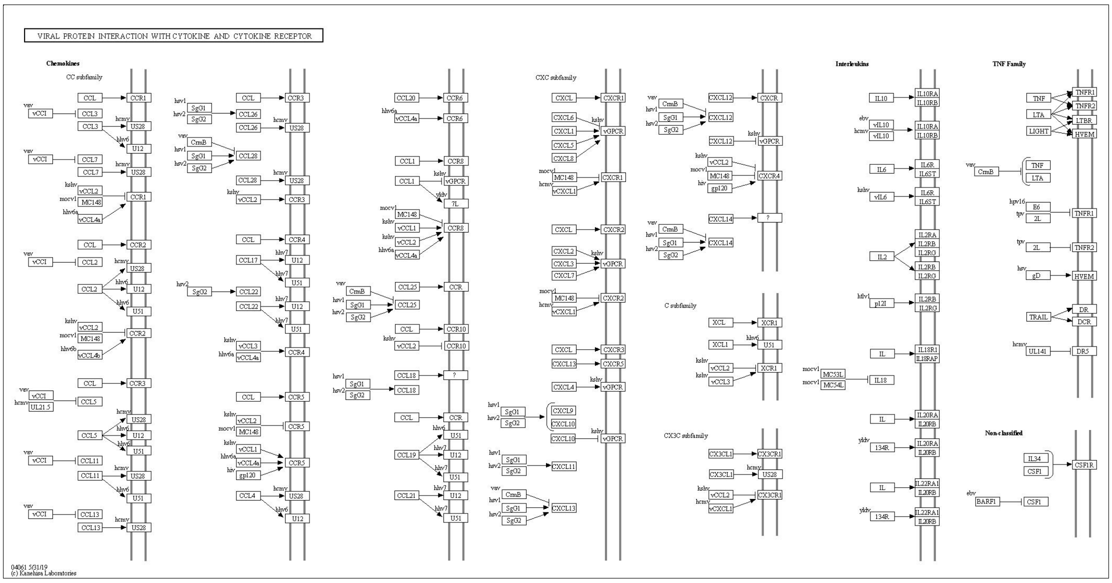

Figure S18 Viral protein interaction with cytokine and cytokine receptor pathway.

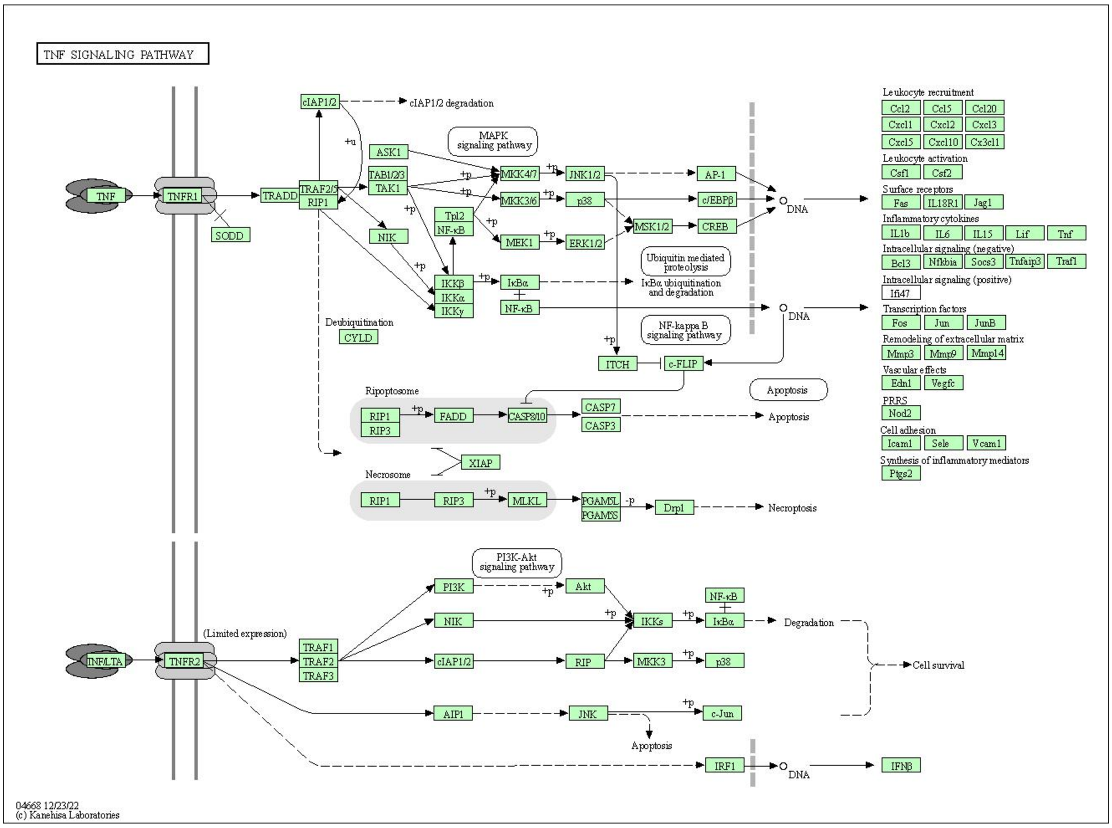

Figure S19 TNF signaling pathway.

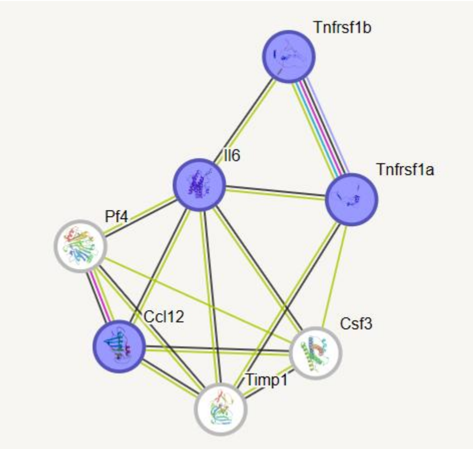

Figure S20 Relationship of differential cytokines in the RSV group compared to the control group (Purple: cytokine enriched in TNF signaling pathway).

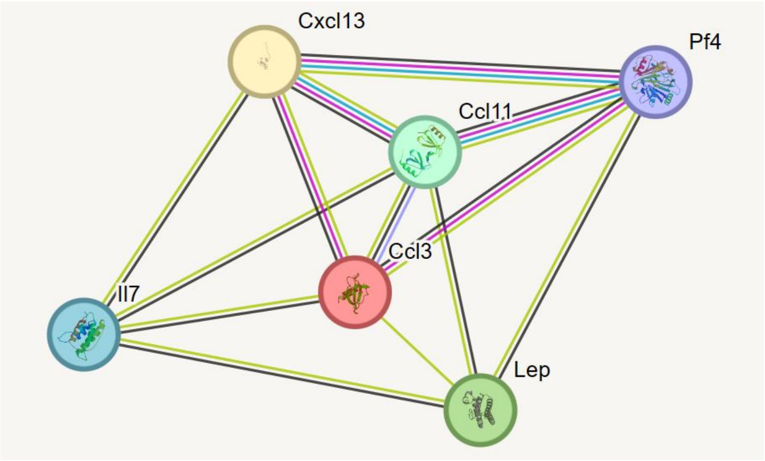

Figure S21 Relationship of differential cytokines in the ZTOI group compared to the RSV group

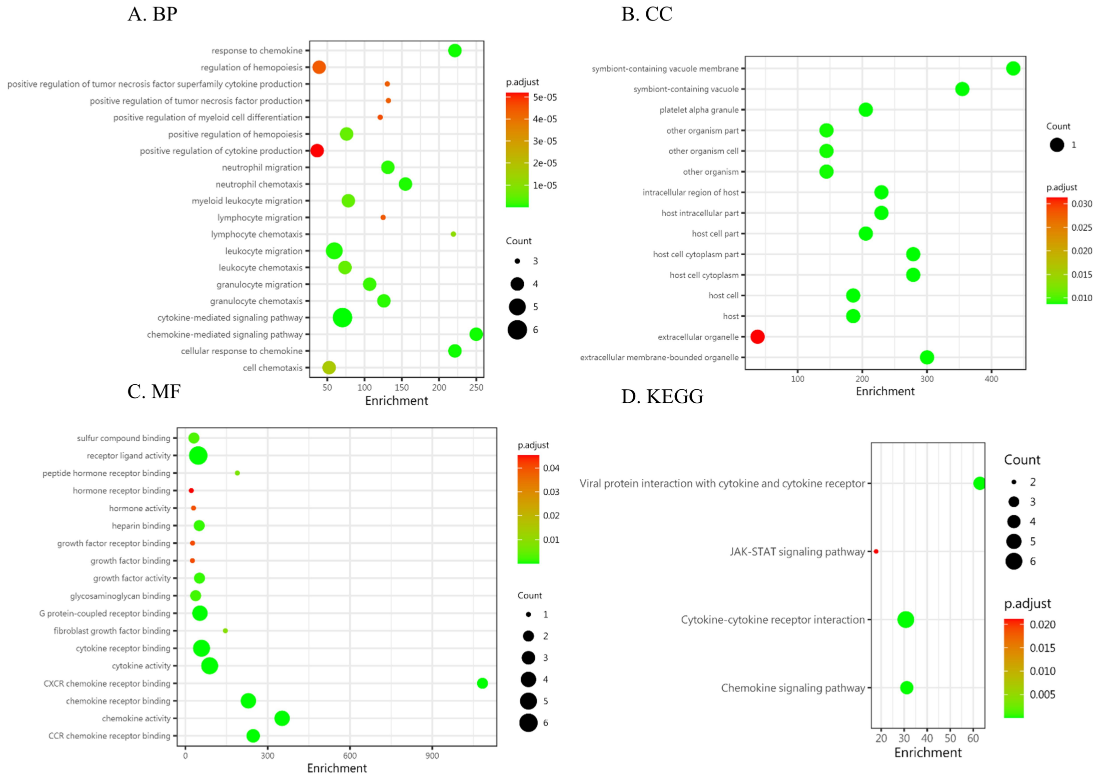

Figure S22 Differential cytokines analysis of the ZTOI-H group compared to the RSV group.

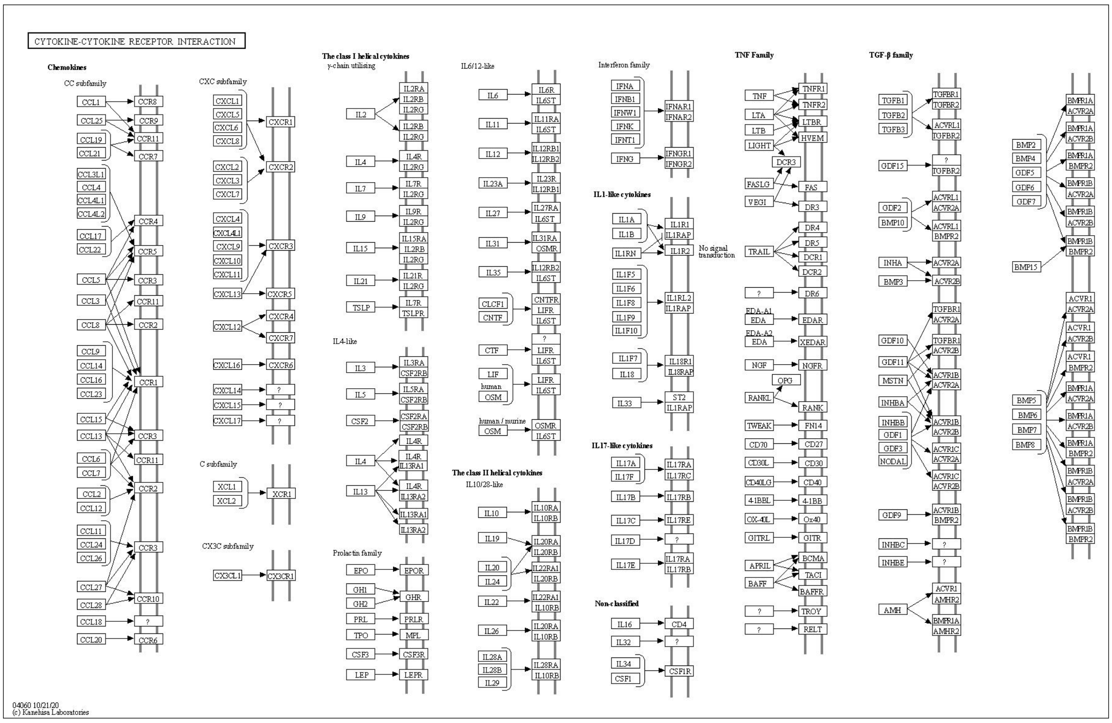

Figure S23 Cytokine-cytokine receptor interaction.

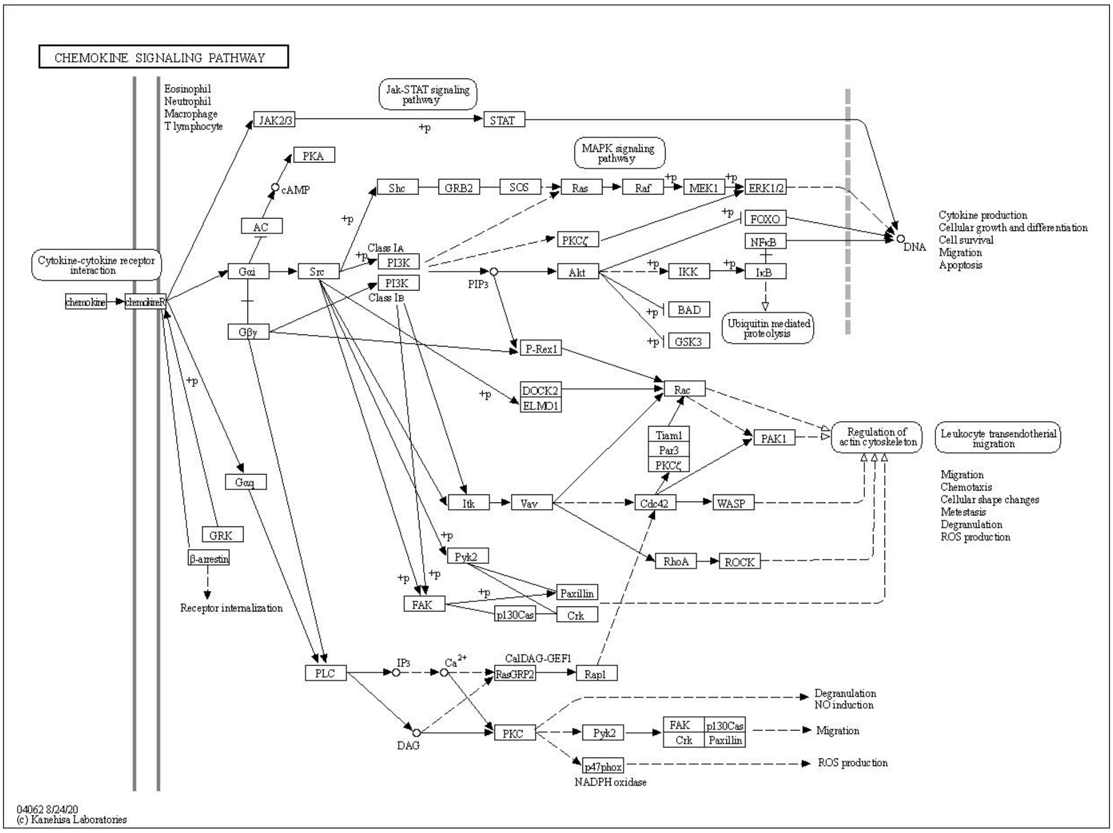

Figure S24 Chemokine signaling pathway.

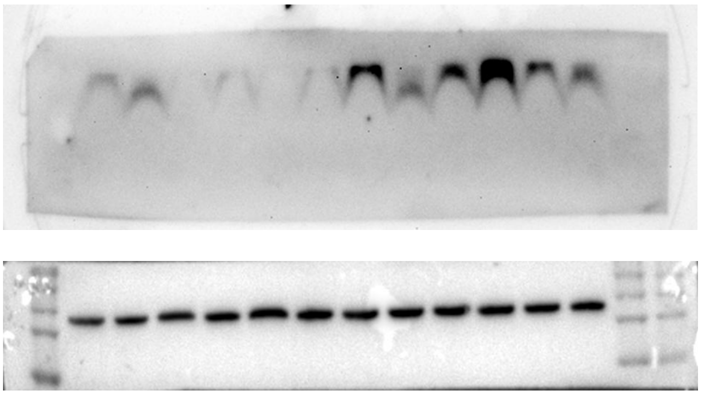

Figure S25 Original western blot figures of figure 5A.
